# Gene Expression Dynamics in *Monilinia fructicola* and *Prunus persica* During the Battle for Infection Revealed by Dual RNA-Seq Analysis

**DOI:** 10.3390/jof12100738

**Published:** 2026-10-01

**Authors:** Domenico Di Cosmo, Mario E. E. Franco, Marco Crudele, Donato Gerin, Lucia Landi, Stefania Pollastro, Francesco Faretra, Rita Milvia De Miccolis Angelini

**Affiliations:** 1 Department of Soil, Plant and Food Sciences, University of Bari Aldo Moro, 70126 Bari, Italy; domenico.dicosmo@uniba.it (D.D.C.); marioemilioernesto.franco@uniba.it (M.E.E.F.); marco.crudele@uniba.it (M.C.); donato.gerin@uniba.it (D.G.); stefania.pollastro@uniba.it (S.P.); francesco.faretra@uniba.it (F.F.); 2 Department of Agricultural, Food and Environmental Sciences, Marche Polytechnic University, 60131 Ancona, Italy; l.landi@univpm.it

**Keywords:** brown rot, transcriptomics, functional networking, peach, molecular plant pathology

## Abstract

Brown rot, caused by *Monilinia fructicola*, poses a significant threat to global peach production, but the molecular interactions underlying host defence and pathogen colonisation remain incompletely defined. In this study, we used dual RNA-Seq to simultaneously capture the gene expression profiles of *Prunus persica* and *M. fructicola* during early infection (12 and 24 h post-inoculation). High-depth sequencing enabled a detailed analysis of transcriptional dynamics in both organisms. *P. persica* displayed a delayed yet robust defence response with marked activation of immune signalling, oxidative burst, cell wall fortification, hormone signalling, and secondary metabolism at 24 hpi. *M. fructicola* initiated infection rapidly, first expressing cell wall-degrading enzymes, then upregulating protein synthesis, energy metabolism, effector proteins, phytotoxins, and stress adaptation genes. Integrated analysis revealed a tightly coordinated interplay between fungal virulence strategies and peach defence mechanisms, including cell wall modification and immune responses. These findings provide a comprehensive framework for host–pathogen dynamics, offering actionable insights for developing resistant peach cultivars for field cultivation and for targeted strategies to improve post-harvest disease management and fruit shelf-life.

## 1. Introduction

Brown rot, caused by *Monilinia* spp., is a globally significant disease affecting stone and pome fruits, resulting in substantial pre- and postharvest losses and persistent challenges for integrated disease management [1]. *Monilinia fructicola* (G. Winter) Honey is recognised as the most aggressive species among the etiological agents, and its expanding distribution poses a major threat to peach (*Prunus persica* L.) production in Europe and worldwide [2,3,4]. Disease control depends on integrated strategies, including cultural practices, fungicide applications, and ongoing research into host resistance and pathogen biology. However, the molecular mechanisms underlying fungal infection and host defence remain incompletely understood despite the disease’s economic significance.

*M. fructicola* is a necrotrophic fungus that colonises host tissues by manipulating plant cell death and deploying diverse pathogenicity factors. Its infection strategy involves orchestrated secretion of carbohydrate-active enzymes (CAZymes), proteases, effector proteins, transporters, and phytotoxic secondary metabolites that collectively promote tissue maceration, nutrient acquisition, and suppression of host immunity [5,6,7]. Like other *Sclerotiniaceae*, *Monilinia* species exhibit an initial asymptomatic phase before extensive necrotrophic growth, indicating that disease development results from a complex molecular interplay between the pathogen and the host rather than simple tissue destruction [8,9,10].

Recent advances in sequencing technologies have substantially deepened our understanding of *Monilinia* biology. Whole-genome and comparative genomic analyses have identified both conserved and species-specific candidate effectors, secreted proteins, CAZymes, transporters, and secondary metabolite biosynthetic gene clusters implicated in pathogenicity and host adaptation [11,12,13,14,15,16,17,18]. Functional studies have validated the roles of several virulence-associated genes, including those regulating oxidative stress tolerance and effector proteins that are differentially expressed during peach infection [19].

Evidence from recent functional and genetic studies increasingly indicates that the pathogenicity of *M. fructicola* is closely linked to specific host responses. During colonization of fruit, *M. fructicola* acidifies peach and nectarine tissues via gluconic acid production; these altered pH conditions enhance the expression of fungal polygalacturonases and facilitate pathogen-induced modification of the host environment, ultimately promoting cell-wall degradation [20]. In addition, genetic analyses of peach populations have identified QTLs and candidate genes implicated in the recognition of *M. fructicola* and the subsequent activation of both PAMP-triggered and effector-triggered immunity [21]. Most recently, functional assays of *M. fructicola* candidate effectors demonstrated that several proteins could induce cell death in *Prunus* tissues, providing compelling experimental evidence for the involvement of fungal effectors in modulating host cell viability during infection [22].

In parallel, transcriptomic and metabolomic studies have expanded our understanding of peach defence responses to *M. fructicola*. Integrated omics approaches demonstrate that infection triggers widespread transcriptional reprogramming, affecting jasmonic acid, salicylic acid, and calcium signalling pathways, phenylpropanoid and flavonoid biosynthesis, reactive oxygen species homeostasis, and cell-wall reinforcement [23,24,25,26]. Comparative analyses of resistant and susceptible cultivars have revealed coordinated defence networks involving lignin deposition, anthocyanin accumulation, key transcription factors (WRKY and MYB families), and receptor-mediated immune signalling [25,26]. Further transcriptomic studies of defence-inducing treatments, such as chitosan and *Bacillus amyloliquefaciens*, highlight the activation of pathogen recognition, hormone signalling, and cell wall remodelling pathways that restrict fungal colonisation [27,28].

Despite these advances, most studies have investigated fungal virulence and host defence in isolation or have focused primarily on induced resistance mechanisms, with few examining both organisms simultaneously. Genome-wide analyses capturing transcriptional responses in both host and pathogen remain limited. To date, the only comprehensive dual RNA sequencing (RNA-Seq) study in the *Monilinia* genus examined the *Monilinia laxa*–nectarine interaction, revealing that fruit developmental stage significantly influences both fungal infection strategies and host defence responses [29]. However, similar analyses of the economically important *P. persica*–*M. fructicola* pathosystem are lacking.

Dual RNA sequencing enables simultaneous monitoring of transcriptional changes in both interacting organisms [30], facilitating identification of coordinated regulatory networks underlying disease development. By capturing the molecular dialogue during infection, this approach allows investigation of fungal virulence factors and host defence responses within a unified biological context.

In this study, we employed dual RNA-Seq to characterise the transcriptional landscape of the *P. persica*–*M. fructicola* interaction during fruit infection. Our objectives were to identify fungal genes associated with virulence and host colonisation, characterise peach defence responses, and provide an integrated perspective on the molecular mechanisms driving brown rot disease progression.

## 2. Materials and Methods

### 2.1. Fungal Isolate and Growth Conditions

The monoconidial strain Mfrc123 (CBS 144850) of *M. fructicola* used in this study was originally isolated from *Prunus avium* fruit collected in a commercial orchard in Apulia, southern Italy. Its genome has been previously assembled and characterised [13,14,15], and all sequence data are publicly available through the NCBI database. The isolate was stored in 10% (*v*/*v*) glycerol at −80 °C, then revived and routinely cultured on potato dextrose agar (PDA). PDA was prepared by infusing 200 g peeled and sliced potatoes at 60 °C for 1 h, adding 20 g dextrose, adjusting pH to 6.5, and incorporating 20 g agar (Oxoid No. 3; Oxoid Ltd., Basingstoke, UK) per litre. For conidia production, cultures were incubated on PDA at 25 ± 1 °C in darkness for 7 days (Figure S1).

### 2.2. Fruit Inoculation, Disease Progression Assessment, and Sample Collection

Healthy *P. persica* cv. Tirrenia fruits were surface sterilised by immersion in 2% (*v*/*v*) sodium hypochlorite for 2 min, rinsed twice with sterile distilled water, and air-dried at room temperature. Each fruit was wounded with a sterile needle to a depth of 5 mm and inoculated with 50 μL of a conidial suspension (1 × 10^6^ conidia mL^−1^) of *M. fructicola*. The suspension was prepared from 7-day-old cultures by gently scraping the surface of each colony with a sterile metal inoculating loop in the presence of 3 mL sterile distilled water with 0.01% (*v*/*v*) Tween 20 (Sigma-Aldrich, St. Louis, MO, USA). The resulting suspension was filtered through glass fiber-fitted Pasteur pipettes to remove mycelial fragments. The concentration of conidia was determined and adjusted using a hemocytometer. Six inoculation points were made per fruit. Fruits were placed on sterilised Nestipacks (Nespak, Massa Lombarda, Italy) and incubated in a moist chamber at 25 ± 1 °C in the dark. Control fruits were mock-inoculated with sterile distilled water containing 0.01% (*v*/*v*) Tween 20.

Samples were collected at 0, 12, and 24 h post-inoculation (hpi), representing pre-symptomatic and symptomatic stages of infection. This sampling strategy captured gene expression dynamics associated with the onset of visible disease.

Lesion diameters were analysed using the Kruskal–Wallis test to assess differences among sampling times. The six inoculation sites on each peach were treated as technical subsamples, and their mean lesion diameter was calculated to yield a single value per fruit. Three independent fruits served as biological replicates for each sampling time (*n* = 3). When the Kruskal–Wallis test indicated significant differences, pairwise comparisons were conducted using Dunn’s post hoc test with Holm correction for multiple comparisons. Statistical significance was defined as *p* < 0.05.

Tissue samples were collected from three fruits per biological replicate (in total, three independent replicates) using a sterile cork borer to excise a fixed-size plug (5 mm deep, 10 mm diameter) centered on the inoculation site. This ensured inclusion of the inoculation site and any associated decayed tissue in each sample. The excised tissues were combined, immediately flash-frozen in liquid nitrogen, and stored at −80 °C until further processing.

### 2.3. RNA Extraction, Library Preparation, and Illumina Sequencing

Total RNA was extracted from peach fruit (*P. persica*) using a modified cetyltrimethylammonium bromide (CTAB)-based protocol [31]. Tissue was ground in liquid nitrogen, and 200 mg of the resulting powder was immediately mixed with 1 mL of pre-warmed extraction buffer (Food Lysis Buffer; Qiagen, Milan, Italy). The mixture was incubated at 65 °C for 15 min with intermittent vortexing. RNA was extracted with an equal volume of chloroform–isoamyl alcohol (24:1, *v*/*v*). After centrifugation at 12,000× *g* for 20 min, the supernatant was transferred to a new tube and re-extracted with chloroform–isoamyl alcohol (24:1, *v*/*v*). After a second centrifugation (12,000× *g* for 20 min), 0.25 volumes of 10 M LiCl were added to the supernatant, then incubated overnight at 4 °C to precipitate RNA. The mixture was centrifuged at 12,000× *g* for 35 min at 4 °C, and the pellet was resuspended in 500 μL SSTE buffer (1 M NaCl, 0.5% SDS, 10 mM Tris-HCl [pH 8.0], 1 mM EDTA [pH 8.0]). Samples were re-extracted with an equal volume of chloroform–isoamyl alcohol (24:1, *v*/*v*), centrifuged at 14,000× *g* for 10 min at 4 °C, and two volumes of 100% ethanol were added to the aqueous phase. RNA was precipitated at −80 °C for 30 min, centrifuged at 14,000× *g* for 20 min at 4 °C, washed with 0.1 mL of 75% ethanol, air-dried, and dissolved in RNase-free water.

RNA quality and concentration were assessed using a NanoDrop 2000 spectrophotometer (Thermo Fisher Scientific, Wilmington, DE, USA) and a Qubit 2.0 fluorometer (Life Technologies, Paisley, UK). RNA integrity was evaluated with a 2100 Bioanalyzer (Agilent Technologies, Santa Clara, CA, USA). All RNA samples were stored at −80 °C until use. Fifteen cDNA libraries were prepared, corresponding to three biological replicates for each experimental condition across the three time points (0, 12, and 24 hpi for inoculated fruits, and 12 and 24 hpi for uninoculated fruits).

cDNA libraries were prepared using the TruSeq Stranded mRNA LT Sample Prep Kit (Illumina, San Diego, CA, USA), and 2 × 100 bp paired-end sequencing was performed on a NovaSeq 6000 platform (Illumina) by Macrogen Inc. (Next-Generation Sequencing Service, Geumcheon-gu, Seoul, Republic of Korea). The generated raw reads were uploaded to NCBI’s Sequence Read Archive (BioProject accession: PRJNA1519183).

### 2.4. Data Analysis for P. persica and M. fructicola

Sequencing quality was assessed with FastQC (Andrews, 2010), and reads were trimmed and mapped to the reference genomes.

Sequencing reads for peach were aligned to the PLov2-2N reference genome of *P. persica* cv. Lovell (GenBank assembly accession GCA_000346465.2) using CLC Genomics Workbench 26.0.4 (QIAGEN, Aarhus, Denmark) with default parameters. Expression levels for 26,411 peach genes were quantified as Reads Per Kilobase per Million mapped reads (RPKM), with genes having RPKM values ≥ 0.02 considered expressed. Differential gene expression analysis was performed using edgeR [32], applying stringent parameters for False Discovery Rate (FDR) estimation (estimateCommonDisp value of 1 × 10^−14^). Genes with a fold change (FC) ≥ |2| and FDR ≤ 0.05 in inoculated versus uninoculated peach fruits at 12 and 24 hpi were classified as differentially expressed genes (DEGs). Functional analysis of DEGs was performed in OmicsBox v3.5.4 (BioBam Bioinformatics S.L., Valencia, Spain) [33]. Putative biosynthetic gene clusters (BGCs) were predicted with plantiSMASH version 2.0 [34] using default parameters, integrating predictions with transcriptomic data. Metabolic overview maps and pathway analyses, including secondary metabolism, cellular responses and regulation, jasmonic acid synthesis in hormone signalling, biotic stress pathways, and receptor-like kinases (Ppersica_139, Phytozome v9.0), were generated with MapMan v4 [35].

For *M. fructicola*, sequencing reads were aligned to the fungal reference genome (strain Mfrc123, GenBank assembly accession GCA_008692225.1) [15] using CLC Genomics Workbench with default parameters, and expression levels for all 12,118 annotated fungal genes were quantified as described for peach. Differential gene expression analysis identified *M. fructicola* genes with FC ≥ |2| and FDR ≤ 0.05 in pairwise comparisons among sampling times (12 vs. 0 hpi, and 24 vs. 0 hpi). Samples collected at 0 hpi served as the baseline condition immediately after inoculation to evaluate fungal transcriptional dynamics. These DEGs were functionally analysed using published gene annotations [14]. Putative BGCs were predicted with antiSMASH fungal version 8.0 [36] using default parameters. Effector gene candidates were identified with EffectorP, https://effectorp.csiro.au (accessed on 12 June 2026), combining outputs from EP 1.0 [37], EP 2.0 [38], and EP 3.0 [39] to maximise prediction accuracy and minimise false positives.

Both *P. persica* and *M. fructicola* genomes were further annotated using dbCAN v5.2.9 and eggNOG-mapper v2.1.15 [40] with the eggNOG DB v5.0.2 [41]. Gene Ontology (GO) and Kyoto Encyclopedia of Genes and Genomes (KEGG) pathway enrichment analyses were performed in RStudio 2024.12.1 (Build 563) using the clusterProfiler v4.16.0 package [42], including both over-representation analysis (ORA; FDR < 0.05 and |log2FC| > 1) and gene set enrichment analysis (GSEA; FDR < 0.05). Data processing and visualisation were performed with the dplyr v1.2.1 [43], enrichplot v1.28.4 [42], ggplot2 v4.0.3 [44], and stringr v1.6.0 [45] packages. KEGG pathway visualisation was conducted using the pathview v1.48.0 package [46], utilising KEGG Orthology (KO) identifiers and differential expression results to represent gene-level changes within enriched pathways.

Intra- and interspecific functional networks of upregulated genes, as well as enriched GO terms and KEGG pathways, were inferred through an extensive literature review, enabling contextualisation and validation of the bioinformatic findings.

## 3. Results

### 3.1. Disease Progression

**Figure 1:**
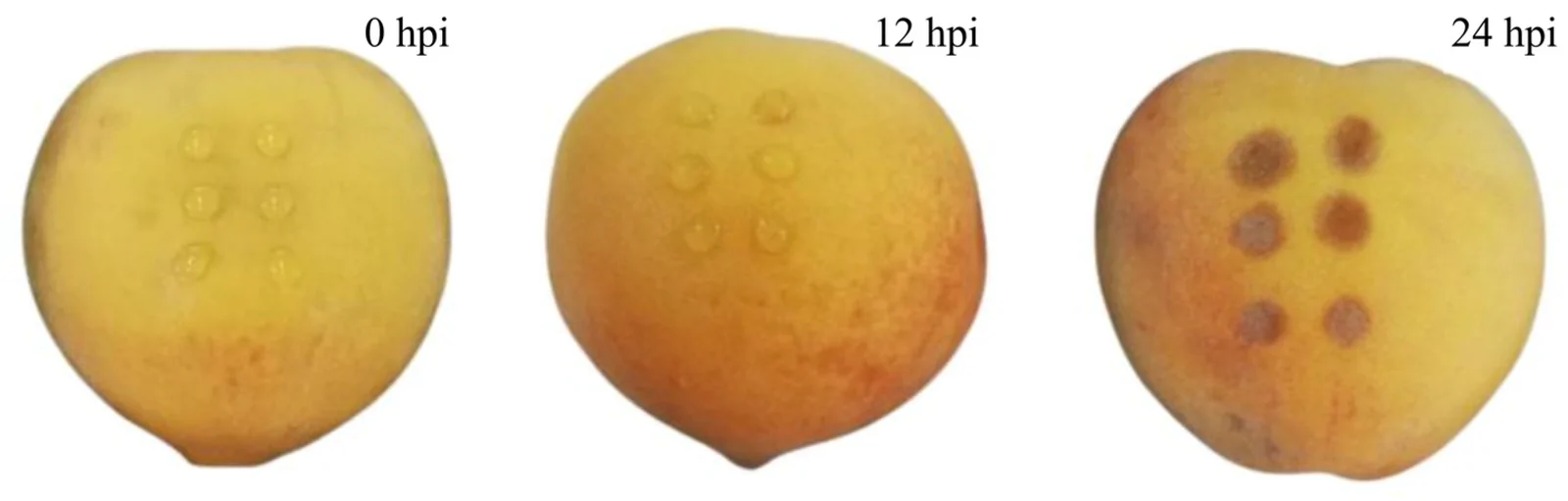
Peach fruits inoculated with a conidial suspension of *M. fructicola* strain Mfrc123 at 0, 12, and 24 h post-inoculation (hpi).

Lesions were not visibly detectable at either 0 or 12 hpi. No symptoms were observed on control fruit at any time point. In contrast, brown lesions became clearly visible at 24 hpi, indicating that the onset of symptom development occurred between 12 and 24 hpi (Figure 1, Table S1). Correspondingly, lesion diameters differed significantly among sampling times (Kruskal–Wallis test, H = 7.62, df = 2, *p* = 0.022). Lesions at 24 hpi were significantly larger than those at both 0 hpi (Dunn’s post hoc test with Holm correction, *p* = 0.0336) and 12 hpi (*p* = 0.0336), whereas no significant difference was observed between 0 and 12 hpi (*p* = 1.000).

### 3.2. Sequencing Data and Read Mapping

Key sequencing statistics are provided in Supplementary Table S2. Illumina sequencing yielded over one billion high-quality reads (Q ≥ 30), with 51–100 million reads per library. Mapping rates to the *P. persica* reference genome were high across all samples (≥84.7%), with a modest reduction at 24 hpi (79.9%), corresponding to increased fungal colonisation. Reads mapping to the *M. fructicola* genome increased over time, from 4.7% at 0 hpi to 8.6% at 24 hpi, consistent with fungal biomass accumulation. Overall, mapping efficiency and read distributions were as expected for dual RNA-seq experiments in plant–pathogen systems.

### 3.3. Global Transcriptional Response During Infection

At 12 hpi, before visible symptoms emerged, initial transcriptional changes were detected in both peach and *M. fructicola*. By 24 hpi, when brown rot lesions became apparent, gene expression was extensively remodulated in the host, reflecting the transition to symptomatic infection.

**Figure 2:**
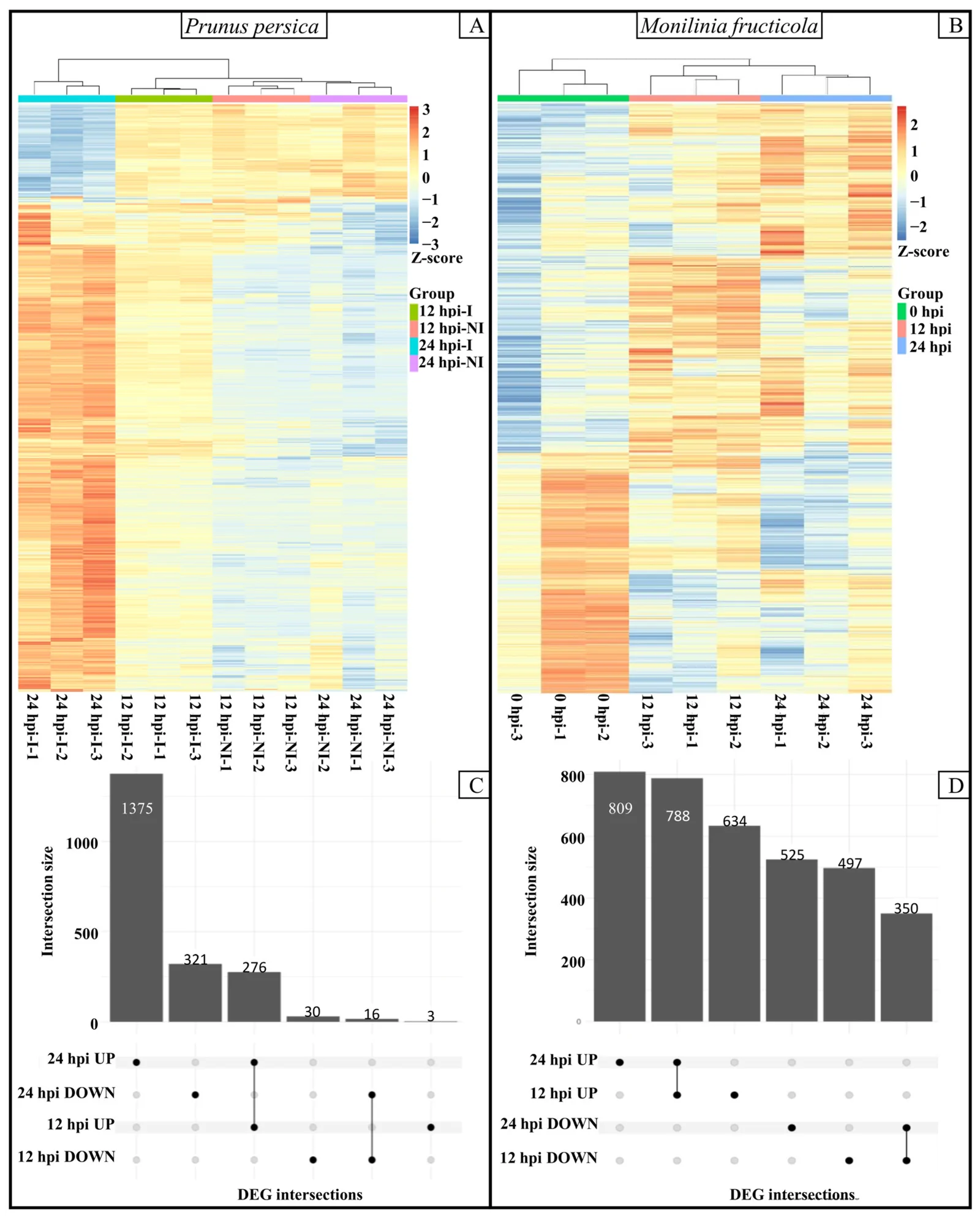
Hierarchical clustering heatmaps and UpSet analysis of differentially expressed genes in *Prunus persica* and *Monilinia fructicola* during infection. (**A**) Hierarchical clustering heatmap of peach DEGs. (**B**) Hierarchical clustering heatmap of *M. fructicola* DEGs. (**C**) UpSet plot showing the overlap of peach DEGs identified at 12 and 24 h post-inoculation (hpi). (**D**) UpSet plot showing the overlap of *M. fructicola* DEGs identified at 12 and 24 hpi. I = inoculated; NI = uninoculated.

Hierarchical clustering of DEGs revealed distinct temporal transcriptional dynamics in both *P. persica* and *M. fructicola* during infection. In *P. persica*, inoculated samples at 24 hpi formed a distinct cluster, separate from both uninoculated samples (12 and 24 hpi) and inoculated samples at 12 hpi (Figure 2A). This shift in gene expression at 24 hpi coincided with the onset of lesion development, indicating that the host response intensifies as symptoms develop (Figure 1). More DEGs were identified at 24 hpi (1988 genes) than at 12 hpi (325 genes), suggesting that the primary host response is triggered later during infection (Figure 2C). The peach response was dominated by gene induction, with approximately 82% of DEGs upregulated (1654 up vs. 367 down).

*M. fructicola* initiated a rapid and robust transcriptional modulation upon host contact. Hierarchical clustering grouped the 12 hpi and 24 hpi samples together, separating them from 0 hpi samples, indicating that a substantial infection-associated transcriptional response was already established by 12 hpi (Figure 2B). Similar numbers of DEGs were detected at 12 hpi (2269 genes) and 24 hpi (2472 genes), suggesting that *M. fructicola* rapidly establishes an infection-specific transcriptional program that is maintained and refined throughout colonisation (Figure 2D). UpSet analysis showed 634 genes specifically induced at 12 hpi, 809 upregulated exclusively at 24 hpi, and 788 overexpressed at both time points, demonstrating distinct transcriptional programs at the two time points. The fungus exhibited a balanced transcriptional response, with 2231 genes upregulated and 1372 downregulated, indicating extensive gene activation and repression.

The complete expression dataset, including RPKM values and differential expression results for both *P. persica* and *M. fructicola*, is provided in Table S3.

### 3.4. Functional Changes During Infection

**Figure 3:**
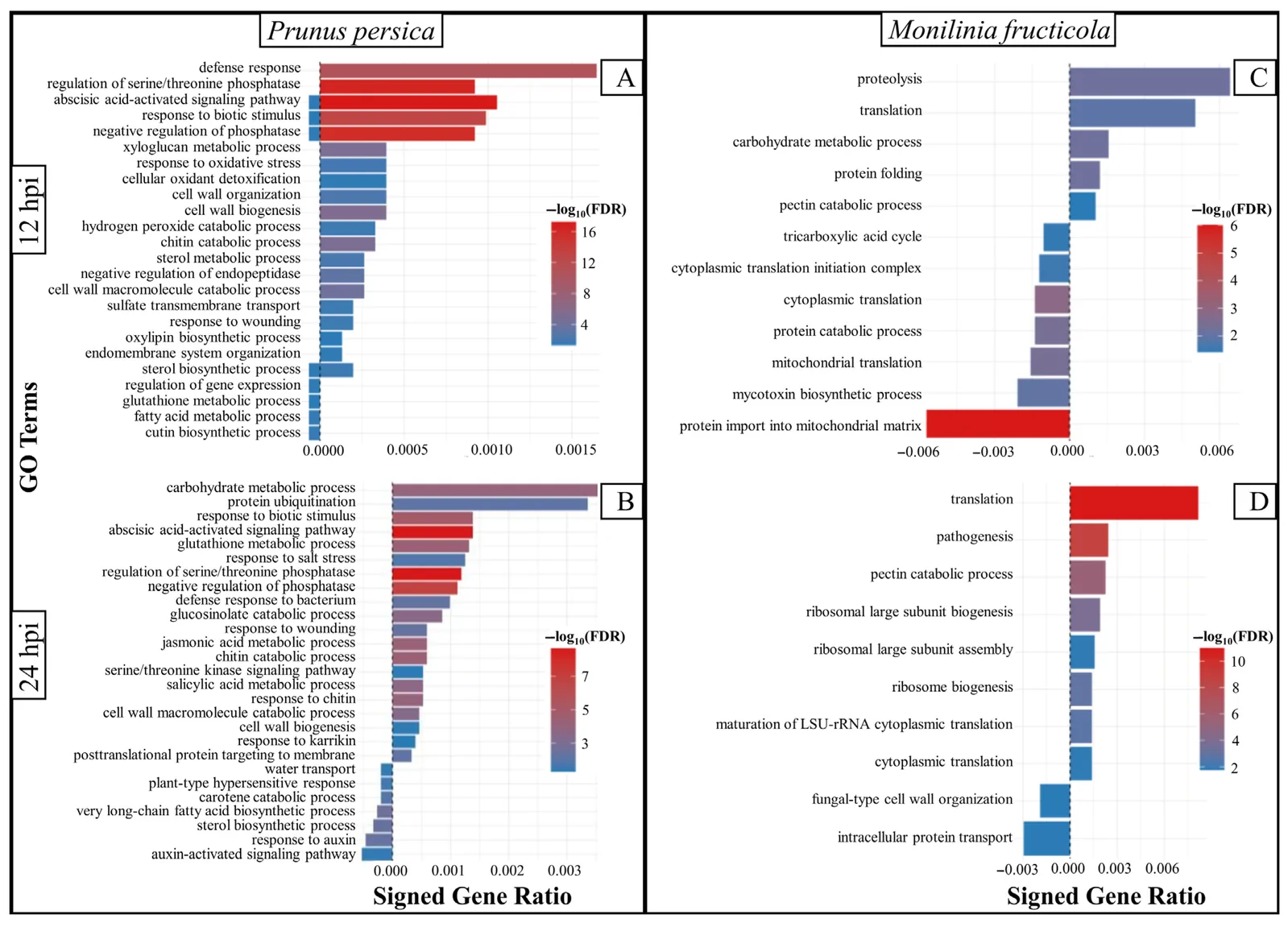
Gene Ontology (GO) biological process enrichment analysis of differentially expressed genes (DEGs) during infection. Bar plots display enriched GO terms at 12 and 24 h post-inoculation (hpi) for *P. persica* (**A**,**B**) and *M. fructicola* (**C**,**D**), respectively. Panels (**A**,**C**) present results at 12 hpi, while panels (**B**,**D**) present results at 24 hpi. The *x*-axis displays the signed gene ratio: GO terms extending to the right of the zero-reference line indicate enrichment predominantly among upregulated DEGs, while those extending to the left indicate depletion, predominantly among downregulated DEGs. Bar colour represents enrichment significance as −log_10_(FDR).

**Figure 4:**
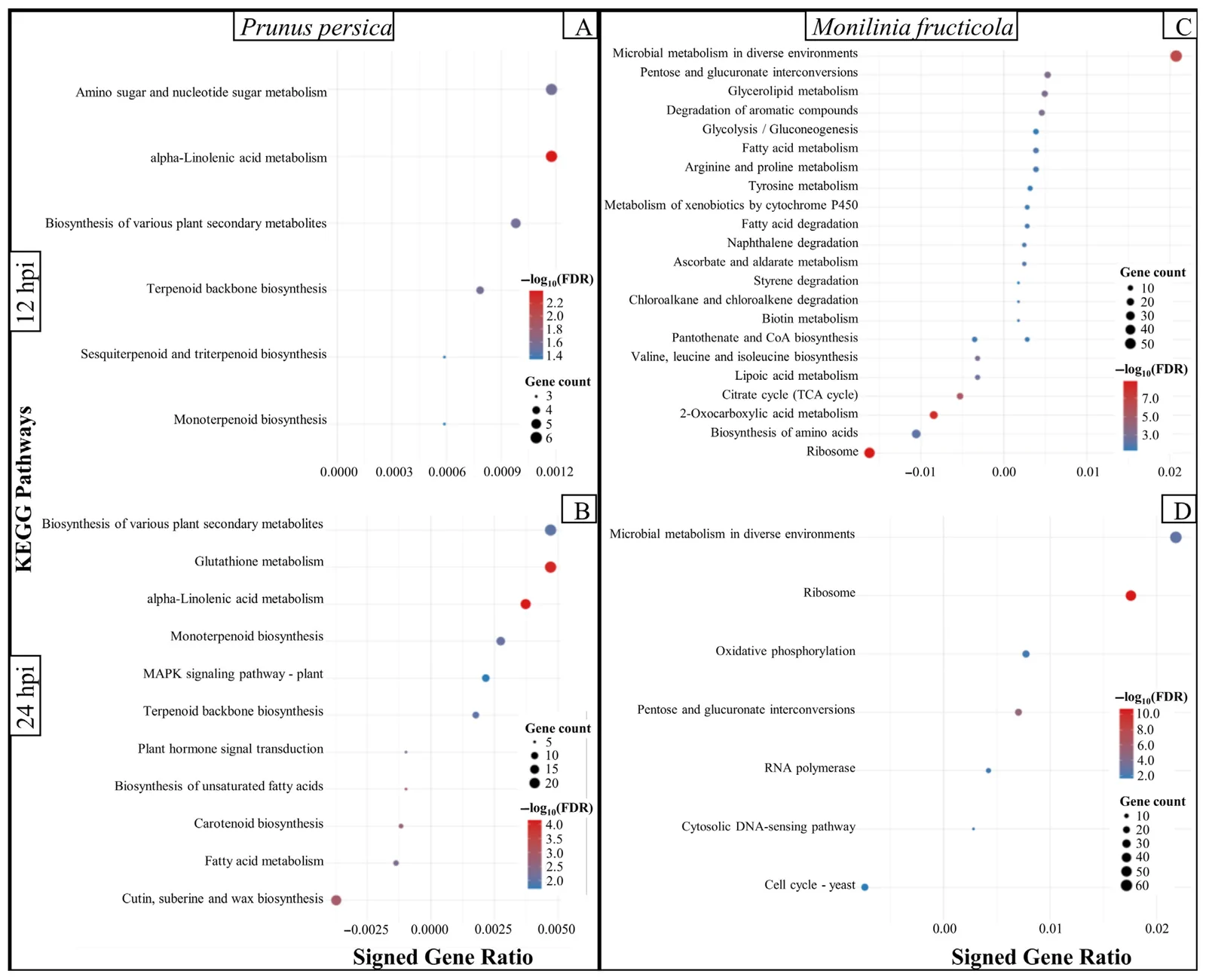
KEGG pathway enrichment analysis of differentially expressed genes (DEGs) during infection. Dot plots illustrate significantly enriched KEGG pathways at 12 and 24 h post-inoculation (hpi) for *P. persica* (**A**,**B**) and *M. fructicola* (**C**,**D**), respectively. Panels (**A**,**C**) present results at 12 hpi, while panels (**B**,**D**) present results at 24 hpi. The *x*-axis shows the signed gene ratio: pathways to the right of the zero-reference line indicate enrichment predominantly among upregulated DEGs, while those to the left indicate depletion predominantly among downregulated DEGs. The distance from zero reflects the magnitude of the signed gene ratio. Dot size is proportional to the number of DEGs mapped to each pathway, and dot colour indicates enrichment significance as −log_10_(FDR).

GO and KEGG pathway enrichment analyses revealed dynamic functional responses in *P. persica* and *M. fructicola* during infection (Figure 3 and Figure 4). In *P. persica*, DEGs were predominantly enriched for processes related to defence activation, which intensified at 24 hpi, consistent with the increased number of DEGs. At 12 hpi, enriched processes included early abscisic acid signalling, regulation of protein phosphatase activity, response to biotic stimuli, cell wall biogenesis and organisation, chitin catabolism, and oxidative stress (Figure 3A). By 24 hpi, the repertoire expanded to include glutathione, jasmonic and salicylic acid metabolism, responses to chitin and wounding, carbohydrate metabolism, and heightened defence against pathogens, reflecting the establishment of a robust immune response (Figure 3B).

KEGG pathway enrichment supported extensive transcriptional reprogramming (Figure 4A,B). At both 12 and 24 hpi, pathways involved in secondary metabolism and terpenoid biosynthesis—including terpenoid backbone and mono-, sesqui-, and triterpenoid biosynthesis—were significantly enriched. The pathway “biosynthesis of various plant secondary metabolites” was consistently overrepresented and featured the enzyme scopoletin glucosyltransferase (EC 2.4.1.128), which catalyses the conversion of scopoletin to scopolin. By 24 hpi, further enrichment of glutathione metabolism and mitogen-activated protein kinase (MAPK) signalling suggested intensified detoxification of oxidative stress and immune signalling. In contrast, pathways for plant hormone signal transduction, carotenoid biosynthesis, fatty acid metabolism, and cutin, suberin, and wax biosynthesis were significantly downregulated at this stage.

MapMan overview (Figure S2) further supported these findings, showing widespread metabolic reprogramming during infection. Primary metabolism exhibited activation of cell wall, lipid, and minor carbohydrate metabolism, with repression of photosynthesis, starch and sucrose metabolism, photorespiration, and tetrapyrrole biosynthesis. In secondary metabolism, phenylpropanoid, lignin, simple phenol, and alkaloid biosynthesis were predominantly induced, while wax biosynthesis remained suppressed. MapMan indicated activation of the mevalonate pathway, linked to sterol, sesquiterpene, and triterpene biosynthesis, and repression of the plastidial non-mevalonate pathway involved in carotenoid, phytol, mono-, and diterpene biosynthesis. Biotic stress analysis showed coordinated induction of signalling components, transcription factors, pathogenesis-related (PR) proteins, hydrolytic enzymes, detoxification pathways, and heat-shock proteins, particularly at 24 hpi, confirming delayed yet extensive activation of peach defence responses.

*M. fructicola* exhibited rapid functional modulation immediately after host infection (Figure 3C,D). At 12 hpi, the most enriched GO categories were related to pectin degradation, carbohydrate metabolism, proteolysis, and mycotoxin biosynthesis, highlighting the early onset of necrotrophic growth and host tissue colonisation. By 24 hpi, enrichment shifted toward translation, ribosome biogenesis, ribosomal assembly, and pathogenesis, with persistent representation of pectin catabolism. This pattern indicates ongoing cell wall degradation alongside increased protein biosynthesis, supporting active fungal proliferation.

KEGG pathway analysis substantiated these findings (Figure 4C,D). At 12 hpi, the most enriched pathways were associated with central metabolism—including microbial metabolism in diverse environments, pentose and glucuronate interconversions, fatty acid metabolism, aromatic compound degradation, glycolysis/gluconeogenesis, and xenobiotic metabolism via cytochrome P450—reflecting metabolic adaptations essential for successful host colonisation. By 24 hpi, ribosome biogenesis and oxidative phosphorylation became the most significantly enriched pathways, alongside persistent enrichment of microbial metabolism and pentose and glucuronate interconversions.

### 3.5. Coordinated Activation of Specialised Metabolic Gene Clusters During P. persica–M. fructicola Interaction

Transcriptomic analysis revealed dynamic changes in specialised BGCs during the *P. persica*–*M. fructicola* interaction. Multiple BGCs from both *P. persica* and *M. fructicola* exhibited significant transcriptional modulation across the infection time course, allowing identification of loci with pronounced transcriptional responsiveness.

A total of 45 putative metabolic gene clusters were identified within the *P. persica* genome, including those involved in terpene, polyketide, saccharide, cyclopeptide, alkaloid, and hybrid biosynthetic pathways (Table S4). In the *M. fructicola* genome, 36 gene clusters were detected, comprising polyketide synthase (PKS), nonribosomal peptide synthetase (NRPS), terpene, and hybrid biosynthetic routes (Table S5).

**Figure 5:**
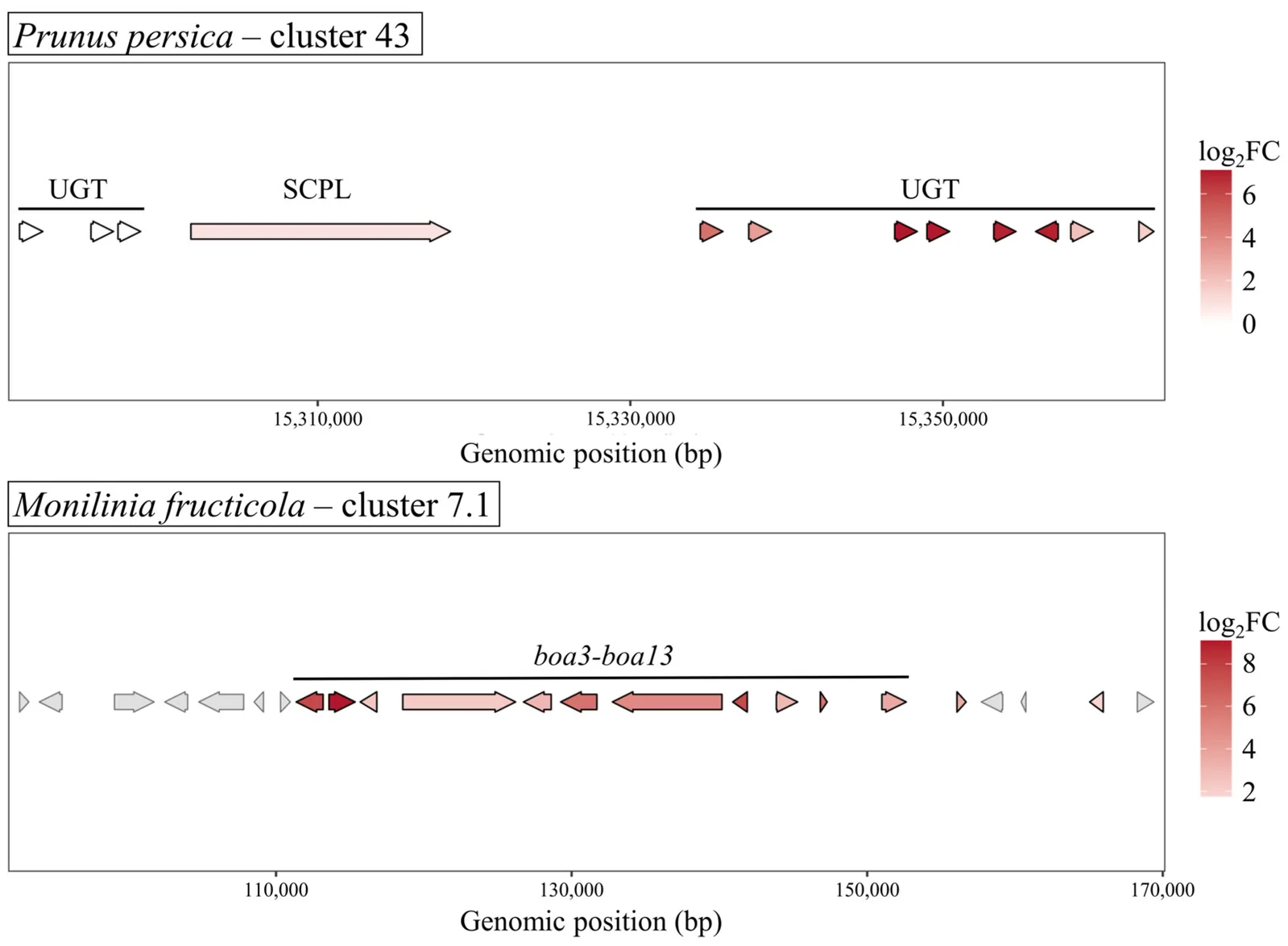
Arrow plots illustrate the genomic organisation and differential expression of *P. persica* cluster 43 at 24 h post-inoculation (hpi) in inoculated versus uninoculated fruits, and *M. fructicola* cluster 7.1 at 24 hpi vs. 12 hpi. Arrow orientation indicates gene direction, while arrow colour indicates log_2_ fold change relative to controls. Grey arrows represent genes with no significant differential expression.

Among the predicted *P. persica* clusters, BGC 43 (chromosome 8, 15,283,061–15,367,472 bp) showed the most robust coordinated transcriptional activation at 24 hpi (Figure 5). This locus encodes several UDP-glycosyltransferases (UGT)—including putative flavonoid 3-*O*-glucosyltransferases, scopoletin glucosyltransferases, and abscisate β-glucosyltransferases—as well as a serine carboxypeptidase-like (SCPL) acyltransferase. These features indicate involvement in glycosylation and structural modification of defence-associated phenolic metabolites.

In *M. fructicola*, BGC 7.1 (scaffold 7, 88,576–170,174 bp) was the most transcriptionally responsive locus. This region corresponds to the phytotoxic polyketide botcinic acid biosynthetic gene cluster, previously identified in *Monilinia* species [15]. Coordinated induction of multiple genes (*boa3*-*boa13*) within this cluster was observed at 24 hpi, consistent with upregulation of botcinic acid biosynthesis during host colonisation (Figure 5).

### 3.6. Major Defence and Pathogenicity Determinants

DEGs implicated in plant defence and fungal pathogenicity were categorised by function, and representative genes with the strongest transcriptional induction were highlighted (Table 1 and Table 2).

In *P. persica*, the most strongly induced genes were associated with oxidative burst, pathogen perception, cell wall reinforcement, antimicrobial protein production, and specialised metabolism. Several berberine bridge enzyme-like (BBE-like) genes exhibited over 3000-fold induction at 24 hpi, underscoring a critical role for oxidative defence mechanisms. Multiple PR proteins, including major allergen Pru ar 1-like and thaumatin-like proteins, were strongly upregulated throughout infection. Enhanced cell wall-associated defence was indicated by increased expression of class V chitinases, β-1,3-glucanases, xyloglucan endotransglucosylase/hydrolases, and polygalacturonase-inhibiting proteins. Upregulation of receptor-like kinases, lectin receptor kinases, ethylene-responsive transcription factors, ACD6, and salicylic acid-binding proteins indicated activation of immune signalling pathways. Genes involved in specialised metabolism, such as β-amyrin synthase and β-amyrin 28-oxidase, were also highly induced, particularly at 24 hpi, supporting the stimulation of antimicrobial secondary metabolite biosynthesis.

*M. fructicola* activated a coordinated pathogenicity program marked by induction of CAZymes, candidate effectors, and stress response proteins. Of 438 identified CAZyme genes, 129 were significantly upregulated during infection (Table S6), including polygalacturonases, carbohydrate esterases, polysaccharide lyases, and auxiliary activity enzymes that facilitate degradation of pectin and other plant cell wall polysaccharides. Effector prediction analysis identified 19 significantly induced candidate effectors (Table S7), including necrosis-inducing Phytophthora protein 1 (NPP1), egh16-like virulence factor, cerato-platanin, and proteins with cyanovirin-N homology (CVNH), fasciclin 1 (FAS1), and FK506-binding protein (FKBP) domains, suggesting a concerted deployment of virulence factors during colonisation. Additional annotation revealed upregulation of further necrosis-inducing proteins and pathogenicity-related genes, as well as stress response proteins such as DAP1, MS95, and Ish1, supporting their role in fungal adaptation to oxidative and chemical stresses during infection.

Collectively, these findings confirm that disease progression involves simultaneous activation of an extensive defence network in *P. persica* and a highly coordinated pathogenicity program in *M. fructicola*. This dynamic interplay—encompassing host cell wall degradation, necrosis induction, stress adaptation, and effector deployment—underscores the complexity of host–pathogen interactions during infection.

### 3.7. Systems-Level Integration of the Host–Pathogen Interaction

To integrate multiple layers of transcriptomic data, we reconstructed functional interaction networks independently for peach and *M. fructicola*.

**Figure 6:**
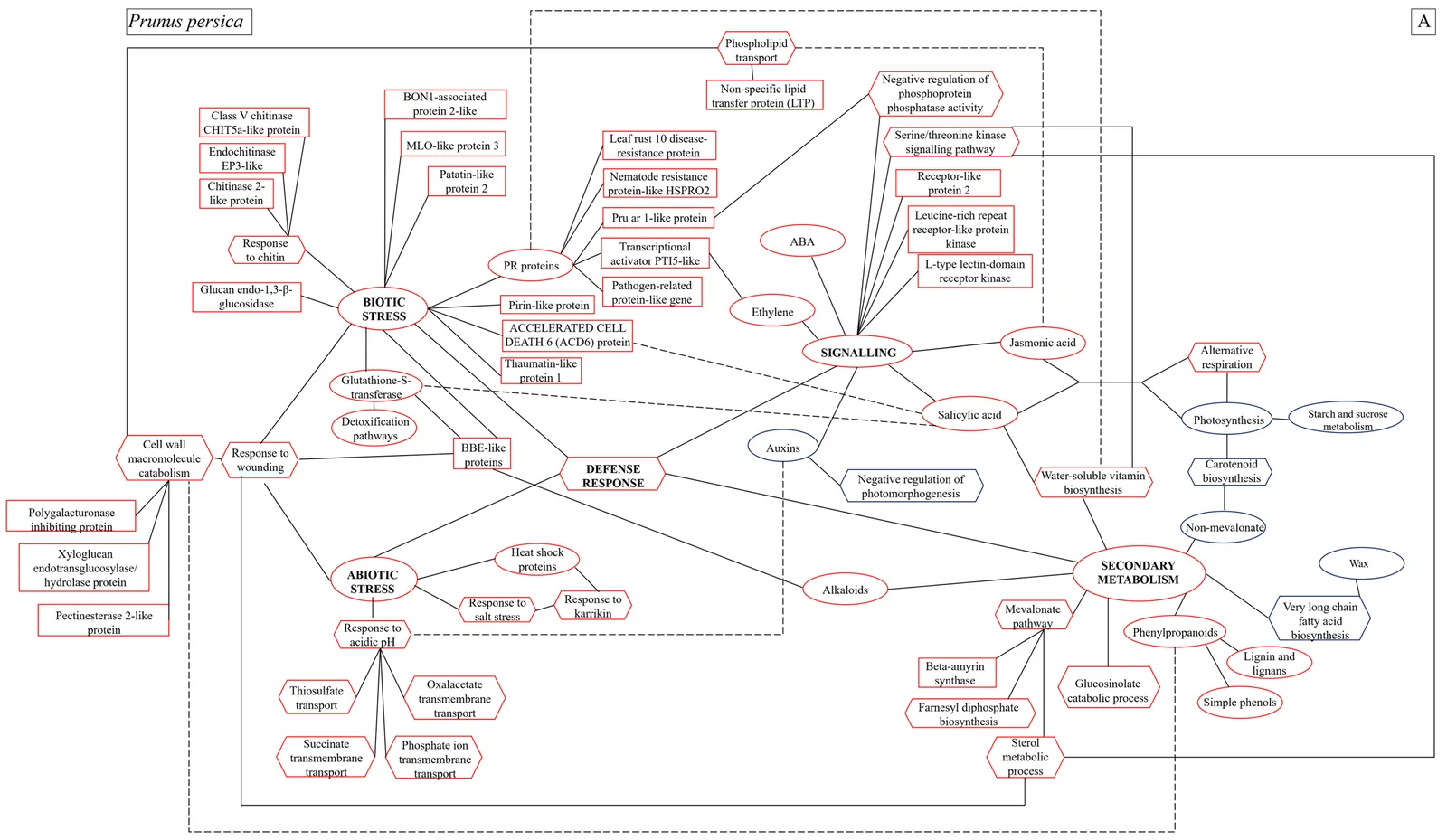

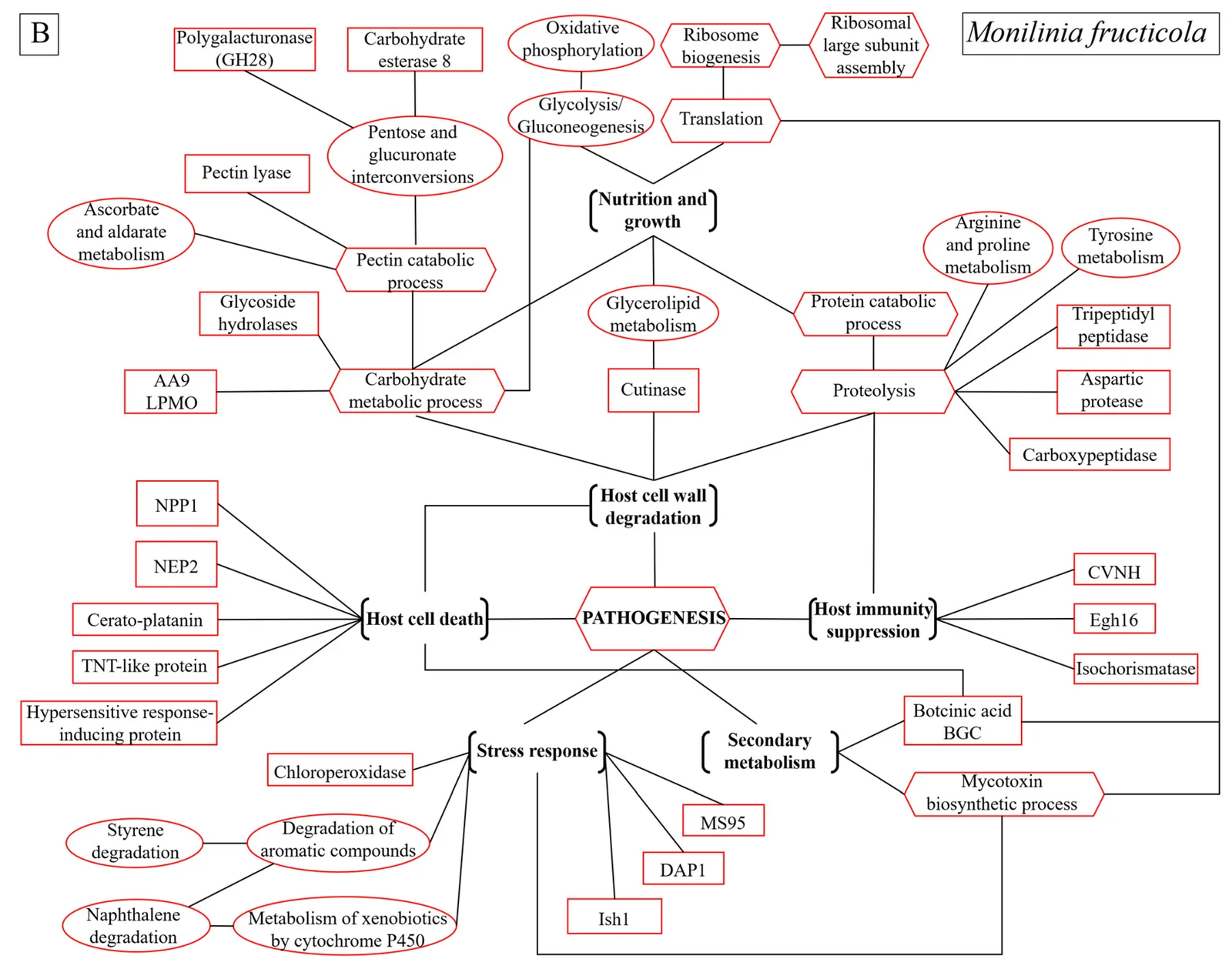
Functional network diagram showing up-regulated (red) and down-regulated (blue) genes (squares), pathways (ellipses), and enriched GO terms (hexagons) in *P. persica* fruits (**A**) and *M. fructicola* (**B**) during infection. This diagram highlights complex host–pathogen interactions at the transcriptomic level.

Within the *P. persica* network, DEGs were connected to enriched GO categories and KEGG pathways, enabling identification of tightly interconnected modules linked to defence responses such as hormone signalling, secondary metabolism, and responses to biotic and abiotic stresses. Similarly, in the *M. fructicola* network, modules related to fungal growth, secondary metabolism, stress adaptation, and pathogenicity were identified (Figure 6).

Within the *P. persica* network (Figure 6A), DEGs mapped to enriched GO categories and KEGG pathways, highlighting a coordinated defence architecture where ‘Biotic Stress’ and ‘Signalling’ connect to downstream execution layers such as ‘Defence Response’, ‘Secondary Metabolism’ and ‘Abiotic Stress’. Similarly, the *M. fructicola* network identifies ‘Pathogenesis’ as the central coordination hub, simultaneously driving core modules dedicated to host cell wall degradation, host cell death, host immunity suppression, and nutrition and growth (Figure 6B).

**Figure 7:**
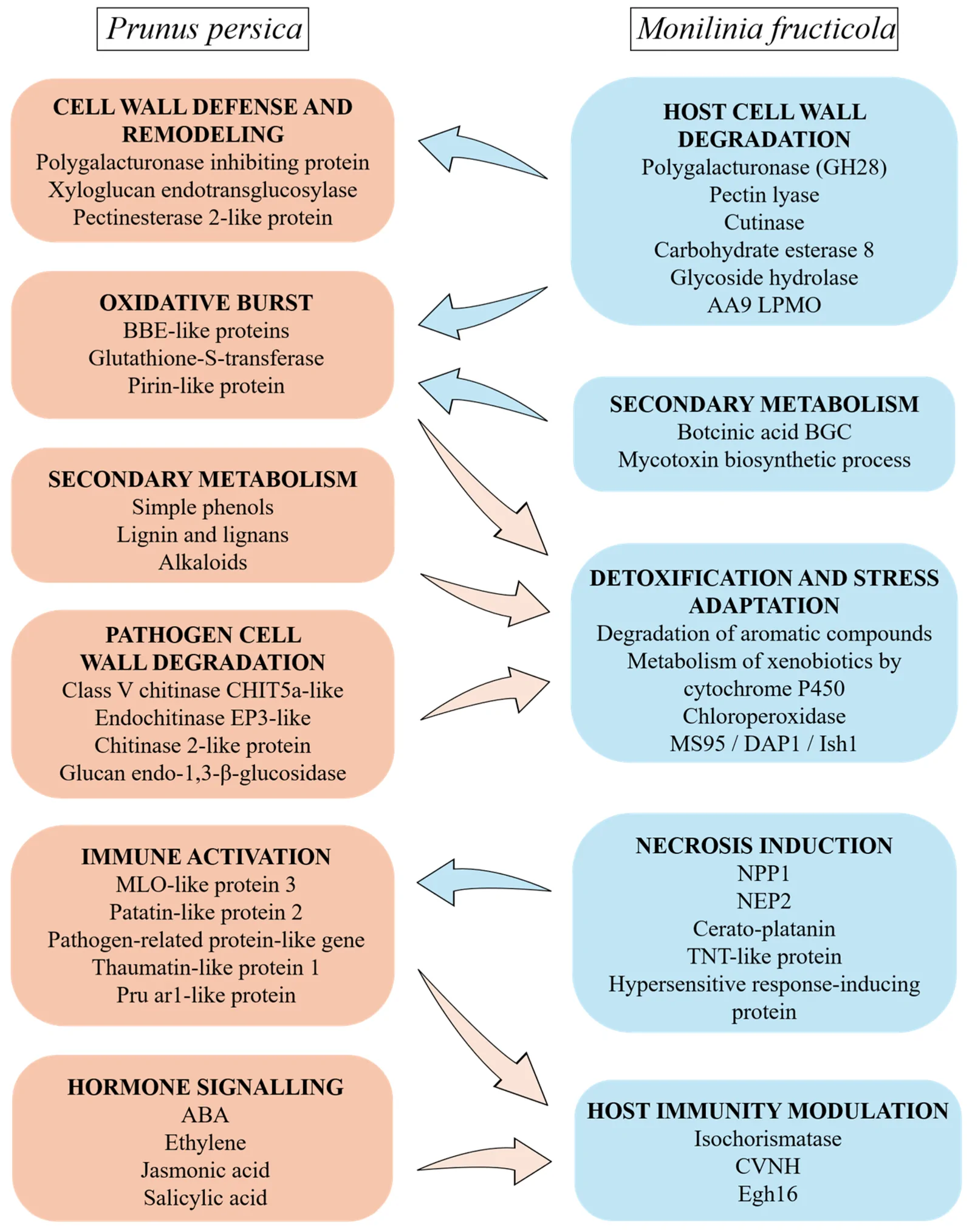
Integrated functional crosstalk between *P. persica* and *M. fructicola* during early infection. The conceptual network summarises the main enriched biological processes GO term, KEGG pathways, virulence-associated genes, and biosynthetic gene clusters identified by dual RNA-Seq analysis in both *P. persica* and *M. fructicola*. Functional modules illustrate coordinated host defence responses and fungal pathogenicity mechanisms. Arrows indicate biologically supported functional relationships inferred from the integrated transcriptomic analyses, emphasising the interplay between plant defence and fungal virulence.

Finally, an integrated host–pathogen interaction network was generated by combining the functional maps of both species (Figure 7). This approach enabled identification of putative biological links between fungal virulence mechanisms and host defence responses. For example, associations were observed between induction of fungal carbohydrate-active enzymes and activation of peach cell wall defence, remodelling, and downstream defence pathways. Activation of plant secondary metabolite biosynthesis and oxidative burst was also compared with transcriptional induction of fungal detoxification pathways and oxidative stress responses.

Collectively, these analyses provide a comprehensive systems-level perspective on the coordinated molecular dialogue underlying the *P. persica*–*M. fructicola* interaction.

## 4. Discussion

This study used dual RNA-Seq to investigate the interaction between *M. fructicola* and *P. persica* fruits, revealing dynamic gene expression changes in both organisms during infection. Our findings elucidate the molecular mechanisms underlying brown rot development and identify the key pathways and factors driving host–pathogen dynamics during fruit infection.

Plants defend against biotic threats through both broad-spectrum and pathogen-specific mechanisms [47], while pathogens deploy counterstrategies to overcome these defences and enhance their virulence [48]. Our dual RNA-Seq analysis pinpoints the central molecular players in this early confrontation between *P. persica* and *M. fructicola*.

Integration of differential gene expression, pathway, and GO analyses identified key defence mechanisms in *P. persica*, notably hormonal signalling, secondary metabolism, and responses to both biotic and abiotic stressors. The defence response in peach was already detectable at 12 hpi and became substantially stronger by 24 hpi, indicating a progressive activation of defence mechanisms as infection progressed.

Early responses involved activation of the glutathione-S-transferase (GST) pathway, induction of PR proteins, and upregulation of ethylene signalling components. GSTs mitigate oxidative stress by detoxifying ROS generated during pathogen attack [49,50], while the induction of PR proteins and ethylene-responsive genes is consistent with the activation of antimicrobial and signalling pathways. The overexpression of the PR protein-associated PTI5-like transcriptional activator, an ethylene-response factor [51], further supports enhanced ethylene signalling during infection. Together, the early induction of GSTs, PR proteins, and hormone-responsive genes indicates that the host rapidly attempts to reinforce defence and control the cellular stress associated with fungal infection.

Receptor-like kinases and lectin-domain kinases were upregulated, supporting their established roles in early pathogen perception and immune signalling [52]. Activation of defence genes included chitinases, glucanases, pectinesterases, xyloglucan endotransglucosylases, and polygalacturonase-inhibiting proteins (PGIPs), suggesting coordinated cell-wall remodelling and reinforcement during infection. PGIPs can restrict fungal pectin degradation and promote further defence activation [53], highlighting cell-wall reinforcement as a key aspect of the peach response. Conversely, genes associated with susceptibility or negative regulation of defence, such as MLO3, BON1, and patatin-like protein 2, were also induced. MLO3 and BON1 are linked to susceptibility or suppression of defence responses, while patatin-like proteins can promote pathogen-associated cell death [54,55,56]. Their induction during *M. fructicola* infection may represent a vulnerable component of the host response. However, functional validation is needed to determine whether they directly contribute to susceptibility in peach. From a practical perspective, these genes are promising candidates for functional studies and, if confirmed, could serve as targets for resistance breeding or gene-editing. The upregulation of Pirin-like proteins and ACCELERATED CELL DEATH 6 (ACD6), along with other biotic stress-associated genes, underscores the complex balance between defence activation and processes that may facilitate disease development.

Upregulation of berberine bridge enzymes (BBEs) and GSTs highlights the importance of coordinated ROS generation and detoxification during the peach response. The induction of BBEs is consistent with recognition of oligogalacturonides (OGs) released during fungal cell-wall degradation, which can act as DAMPs and stimulate defence-associated ROS production, while BBE activity may subsequently contribute to OG turnover and prevent excessive accumulation of these elicitors [57,58]. The concurrent induction of GSTs suggests that the resulting oxidative stress is actively controlled. This balance may be particularly important during infection by a necrotrophic pathogen, for which excessive ROS accumulation and consequent host cell death could ultimately facilitate rather than restrict fungal colonisation. Thus, the coordinated activation of BBE- and GST-associated responses suggests that *P. persica* not only activates ROS-dependent defence signalling but also regulates its intensity to limit excessive tissue damage.

This defence response was accompanied by substantial metabolic reprogramming, characterised by activation of secondary metabolism and concomitant suppression of primary metabolic pathways. The induction of phenylpropanoid metabolism is particularly relevant given the contribution of phenolic compounds to peach resistance against *Monilinia* species [59,60]. Notably, the strong induction of the glycosyltransferase-rich BGC 43 and scopoletin glucosyltransferase indicates that this response extends beyond phenolic biosynthesis to the controlled modification of defence metabolites. Glycosylation can facilitate the storage and transport of phenolic compounds while reducing their phytotoxicity [61], and the conversion of scopoletin to scopolin provides another example of this regulatory strategy [62]. The concomitant induction of the mevalonate pathway, supporting the biosynthesis of sterols, sesquiterpenes and triterpenoids, alongside the downregulation of the MEP pathway and carotenoid biosynthesis, suggests a selective redirection of isoprenoid metabolism towards defence and stress adaptation. This shift is consistent with a broader reallocation of cellular resources from primary metabolism towards defence. Hormonal signalling showed a coordinated temporal pattern during infection. Ethylene- and ABA-associated responses were already evident at 12 hpi, with ABA signalling remaining enriched and ethylene-responsive transcription factors showing further induction at 24 hpi.

In contrast, jasmonic acid (JA) and salicylic acid (SA) signalling became prominent only at 24 hpi. This sequential activation suggests that hormone-mediated responses are dynamically reprogrammed as infection progresses, rather than being activated uniformly from the onset. The early ethylene response is particularly notable, as ethylene signalling has been linked to increased susceptibility during *M. laxa* infection of nectarine by promoting ripening processes [29]. This finding implies that ethylene may serve as a critical interface between host physiology and *Monilinia* infection. The subsequent activation of JA and SA pathways aligns with the progressive intensification of defence responses as fungal colonisation advances.

From an applied perspective, the integrated regulation of oxidative stress, metabolic reallocation, and hormone signalling highlights potential targets for improved brown rot management. Genes and pathways involved in ROS control and defence-associated metabolism could serve as molecular markers in resistance breeding programs. Moreover, the sequential activation of hormone pathways suggests that elicitor treatments could be timed to match infection progression, enhancing their effectiveness.

*M. fructicola* rapidly modulated its transcriptome upon host contact, inducing cell wall-degrading enzymes (CAZymes), proteases, and central metabolic pathways to facilitate tissue colonisation and fungal proliferation. Early infection was marked by extensive activation of enzymes targeting the main structural components of peach tissues, especially pectin, as well as cellulose and the fruit cuticle. Strong induction of GH28 polygalacturonases, carbohydrate esterases, pectate lyases, cutinases, and AA9 lytic polysaccharide monooxygenases, coupled with enrichment of pectin catabolic processes and pentose and glucuronate interconversions, points to a coordinated enzymatic attack on the host cell wall and cuticle. This pattern is consistent with peach tissue composition and supports previous findings that cell wall-degrading enzymes are major virulence factors in necrotrophic fungi [63,64]. Concurrently, robust induction of aspartic proteases, carboxypeptidases, and tripeptidyl peptidases suggests that host protein degradation offers an additional nutrient source and may help inactivate plant defence proteins [65]. The combined degradation of host polysaccharides and proteins provides accessible carbon and nitrogen, in line with the simultaneous upregulation of glycolysis, fatty acid metabolism, and other core metabolic pathways necessary for fungal growth. Notably, pathways related to ribosome biogenesis, assembly, and translation were downregulated early in infection but became among the most significantly upregulated at 24 hpi, indicating a shift from host penetration and tissue degradation to active fungal proliferation and increased protein biosynthetic capacity.

However, successful colonisation requires *M. fructicola* not only to degrade host tissues but also to counteract the defence responses this triggers. In line with this, *M. fructicola* induced candidate effectors involved in plant immunity suppression, including Egh16-like, CVNH, and isochorismatase proteins. Egh16-like effectors are linked to suppression of receptor-associated kinase signalling and early immune activation [66], while CVNH proteins can disrupt ROS-mediated defences, as shown for SsCVNH of *Sclerotinia sclerotiorum*, which interacts with plant class III peroxidase and reduces ROS production and host immune responses [67]. Isochorismatase induction also aligns with interference in salicylic acid-dependent immunity by depleting the isochorismate precursor pool [68]. Thus, *M. fructicola*’s infection strategy integrates physical degradation of host tissue with suppression of multiple layers of plant immunity, targeting cell wall integrity, receptor signalling, ROS production, and salicylic acid-dependent defence.

The concurrent activation of these processes underscores the intricate interplay between fungal virulence and host defence during infection. Degradation of the cell wall by the fungus not only facilitates tissue maceration and nutrient acquisition but also generates signals that trigger peach defence responses, while fungal effectors dampen the resulting immunity. This balance is further shaped by the induction of necrosis-associated proteins and the botcinic acid biosynthetic cluster, which promote host cell death and support the transition to necrotrophic growth. Thus, cell wall degradation, immune modulation, and necrosis induction are not isolated pathogenicity mechanisms but form integrated, interdependent elements of the fungal infection strategy.

Comparison with the mature nectarine–*M. laxa* pathosystem described by Balsells-Llauradó [29] reveals both shared and distinct infection strategies. *M. laxa* exhibited a greater reliance on proteolytic effectors than CAZymes, investing in filamentous growth early in the interaction. In contrast, *M. fructicola* showed robust and coordinated activation of both protein and cell wall degradation, with particularly pronounced induction of pectin-targeting CAZymes. In the present system, transcriptional signatures linked to fungal growth were less evident at early stages and became more prominent by 24 hpi, coinciding with increased ribosome biogenesis, translation, and oxidative phosphorylation. Necrosis-associated proteins and stress-adaptation responses were also strongly induced, indicating an ongoing transition toward active tissue colonisation and adaptation to the host environment. These findings suggest that, despite a shared degradative lifestyle, closely related *Monilinia* species may vary in the timing and relative emphasis of growth, tissue degradation, and pathogenicity mechanisms during colonisation of susceptible *Prunus* fruit.

Throughout infection, *M. fructicola* upregulated genes associated with stress adaptation and detoxification (e.g., DAP1, DDR48, Ish1, cytochrome P450s), supporting survival under oxidative and chemical stress imposed by peach defences. DAP1 encodes a heme-binding protein that regulates ergosterol biosynthesis in response to environmental cues, promoting fungal survival in adverse conditions [69]. The MS95 damage response protein, now referred to as DDR48 (DNA damage response), is involved in oxidative stress resistance, antifungal tolerance, and overall fungal fitness during host colonisation [70]. Similarly, Ish1 is a stress-responsive nuclear envelope protein whose expression is induced by environmental stressors and supports cellular viability during stationary phase, though its precise molecular function remains incompletely characterised [71]. The progressive induction of these genes, along with enrichment of pathways for xenobiotic metabolism by cytochrome P450, degradation of aromatic compounds, and oxidative metabolism, indicates activation of detoxification systems enabling *M. fructicola* to withstand host-imposed stresses. These responses constitute a critical adaptive strategy, allowing sustained fungal growth in the presence of ROS and antimicrobial phenolic compounds.

Dual transcriptomic analysis of host and pathogen elucidated the intricate molecular dialogue underlying brown rot development, revealing a tightly regulated series of reciprocal interactions between fungal virulence strategies and plant defence mechanisms.

Upon fungal penetration, activation of CAZymes and other cell wall-degrading enzymes compromises the structural integrity of the peach cell wall. Fungal polygalacturonases release oligogalacturonides (OGs) from pectin, which serve as damage-associated molecular patterns (DAMPs) that elicit plant immune responses. In turn, *P. persica* swiftly activates PGIPs to detect and inhibit pathogen-derived polygalacturonases, restricting pectin degradation and maintaining OG-mediated defence signalling [72,73]. This interplay drives the simultaneous activation of cell wall defence and remodelling in peach, involving PGIPs, pectin-modifying enzymes, and xyloglucan endotransglucosylase/hydrolases. Moreover, pathogen recognition rapidly triggers an oxidative burst, leading to ROS accumulation that reinforces cell walls and amplifies antimicrobial signalling [74].

Several candidate effector-associated genes provide mechanistic links between specific fungal virulence functions and host defence pathways. In this interaction, *M. fructicola* Egh16-like genes exhibited strong induction at 12 hpi, while MAPK-associated responses in peach became more prominent at 24 hpi. Similarly, expression of the *M. fructicola* CVNH candidate increased from 12 to 24 hpi, mirroring the marked induction of multiple peach peroxidases. These temporal correspondences suggest potential interactions between fungal effector activity and host signalling or ROS homeostasis; however, direct molecular interactions between *M. fructicola* proteins and peach targets have yet to be experimentally demonstrated.

A further potential point of crosstalk is represented by the *M. fructicola* isochorismatase candidate, which exhibited a progressive increase in expression throughout the infection time course. This putative pathogen-mediated interference temporally coincided with a robust activation of SA-associated responses in peach, especially at 24 hpi. These findings are consistent with an antagonistic host–pathogen dynamic: the pathogen may attempt to modulate host SA biosynthesis, while the host responds through compensatory activation of SA-dependent defence pathways, rather than experiencing a complete suppression of immunity.

As colonisation advances, *M. fructicola* appears to intensify its suppression of host defences by upregulating phytotoxin biosynthetic clusters, including those for botcinic acid, as well as necrosis-associated proteins such as NEP2. These molecular changes temporally align with the onset of visible brown lesions, suggesting a role in promoting host cell death and facilitating the pathogen’s transition to necrotrophic growth [75,76]. Nep2-like proteins have been implicated in stimulating ethylene production, reactive oxygen species (ROS) accumulation, and programmed cell death in host tissues [76]. In line with these established activities, the marked late-stage induction of NEP2 was accompanied by elevated expression of ethylene-responsive and ROS-associated defence genes in peach. However, our data do not provide direct evidence of an interaction between NEP2 and specific peach targets. The resulting tissue damage further activates peach defence responses, as demonstrated by the upregulation of receptor-mediated signalling components, defence-related proteins, and hormone-dependent pathways. This likely reflects the plant’s continued attempt to contain disease progression and mitigate pathogen impact [77,78].

At the same time, the host appears to modulate its defence intensity to limit excessive damage, as suggested by the induction of BBE proteins, glutathione metabolism, and glycosylation of phenolic compounds. Conversely, induction of host susceptibility-associated factors, including MLO and patatin-like proteins, may further facilitate cell death and fungal colonisation.

In parallel, *P. persica* reorganises its metabolism by downregulating resource-intensive processes like photosynthesis and reallocating resources toward antimicrobial secondary metabolism.

The resulting interaction can therefore be viewed as a dynamic molecular arms race. As peach defences intensify through ROS production, antimicrobial metabolites and immune signalling, *M. fructicola* responds by activating detoxification and stress-adaptation mechanisms, including P450-mediated xenobiotic metabolism, degradation of aromatic compounds and induction of stress-response pathways. These adaptations reflect the fungus’s growing capacity to withstand host-imposed oxidative and chemical stresses, enabling continued colonisation despite escalating peach defences.

A comparison of the dual RNA-seq results from this study with the targeted RT-qPCR data reported by Landi et al. [19] revealed consistent expression patterns for several *M. fructicola* candidate effectors, including NPP1, NEP2, Egh16, GH28, CVNH, and isochorismatase. Similarly, several peach defence-related genes—such as salicylic acid-binding protein, an ethylene-responsive transcription factor, and a pathogenesis-related protein—also displayed comparable expression patterns between the two studies. Differences in expression magnitude may reflect biological variation among *M. fructicola* isolates and peach cultivars, as well as variation in experimental conditions. Collectively, these findings provide independent and complementary support for the expression patterns of multiple candidate genes identified by dual RNA-seq, while the broader transcriptomic approach highlights the coordinated responses of both host and pathogen during infection. Future work will focus on experimentally validating selected genes identified in this study, using appropriate genetic and functional assays in both *M. fructicola* and *P. persica*.

Overall, this study provides a valuable framework for elucidating the interaction dynamics between *P. persica–M. fructicola*. However, our analysis is confined to the 12–24 hpi time window and thus does not capture transcriptional responses that occur immediately after inoculation or at later stages of disease progression. Increasing temporal resolution in future studies will be essential for identifying early molecular events and for characterizing transcriptional changes associated with advanced stages of necrotrophic development. Comparative analyses across diverse peach cultivars and *M. fructicola* isolates can help identify conserved and genotype-specific responses, while functional validation of key genes and pathways will further clarify their roles in disease development and resistance.

## 5. Conclusions

This study demonstrates the utility of dual RNA-Seq in simultaneously capturing the transcriptional responses of both *P. persica* and *M. fructicola*, providing a comprehensive systems-level perspective on the molecular dialogue underlying brown rot development. By integrating host and pathogen transcriptomes, we reveal that disease progression is governed by a molecular arms race involving fungal cell wall degradation, phytotoxin production, immune modulation, and stress adaptation, intricately linked to host cell wall reinforcement, oxidative burst, immune activation, and specialised metabolism. These findings deepen our understanding of the biological mechanisms that drive the *P. persica*–*M. fructicola* interaction, establish a foundation for future studies on host–necrotrophic pathogen interactions, and identify key molecular pathways and virulence determinants that could inform the development of resistant cultivars and more targeted, sustainable brown rot management strategies.

## Figures and Tables

**Table 1:** Principal *P. persica* defence-associated genes significantly upregulated during *M. fructicola* infection. Log_2_FC values at 12 and 24 hpi are reported.

Functional Category	Gene/Protein	Gene ID	log_2_FC (12 hpi)	log_2_FC (24 hpi)
Oxidative burst and redox homeostasis	BBE-like protein	LOC18779607	6.78	11.75
	BBE-like protein	LOC18793845	6.38	11.26
	BBE-like protein	LOC18778604	4.32	10.11
	BBE-like protein	LOC18780292	3.58	6.07
	BBE-like protein	LOC18778349	3.17	9.89
Immune perception and signalling	PTI5-like transcriptional activator	LOC18780251	2.58	5.75
	HSPRO2-like resistance protein	LOC18784599	2.58	5.49
	Leaf rust 10 disease resistance protein	LOC109946851	2.81	4.39
	MLO-like protein 3	LOC18771951	2.00	7.24
	BON1-associated protein 2-like	LOC18785981	3.32	7.86
	Ethylene-responsive transcription factor	LOC18776415	2.00	5.75
	Non-specific lipid transfer protein (LTP)	LOC18774006	4.17	6.44
	Patatin-like protein 2	LOC18793531	3.91	8.01
	Pirin-like protein	LOC18781479	1.58	4.25
	Pirin-like protein	LOC18793275	1.00	3.17
	Pleiotropic drug resistance protein 2	LOC18772325	2.81	5.81
	ACCELERATED CELL DEATH 6 (ACD6) protein	LOC18779339	3.58	5.86
	ACD6-like protein	LOC18779799	3.32	5.52
	Receptor-like protein 2	LOC18779360	4.32	7.30
	Leucine-rich repeat receptor-like kinase	LOC18768711	2.00	4.17
	L-type lectin-domain receptor kinase	LOC18766594	3.46	7.43
	Salicylic acid-binding protein 2-like	LOC18769532	1.58	5.04
PR proteins and antimicrobial enzymes	Major allergen Pru ar 1-like protein	LOC18790048	4.95	8.21
	Major allergen Pru ar 1-like protein	LOC18793411	4.46	7.31
	Major allergen Pru ar 1-like protein	LOC18790056	3.91	7.07
	Thaumatin-like protein 1	LOC18783179	2.32	5.67
	Pathogenesis-related protein-like	LOC18768372	4.17	8.38
	Class V chitinase CHIT5a-like protein	LOC18789345	4.09	7.00
	Chitinase 2-like protein	LOC18781233	1.00	3.81
	Endochitinase EP3-like	LOC18791427	2.32	4.58
	β-1,3-glucanase	LOC18779277	2.81	2.32
	β-1,3-glucanase	LOC18769272	2.32	8.73
	Polygalacturonase-inhibiting protein (PGIP)	LOC18770913	2.32	4.39
Cell wall reinforcement and remodeling	Pectinesterase 2-like protein	LOC18786681	5.67	9.90
	Pectinesterase/pectinesterase inhibitor	LOC18793253	3.00	8.38
	Xyloglucan endotransglucosylase/hydrolase	LOC18793282	2.58	6.85
Secondary metabolism	β-amyrin synthase	LOC18780496	4.32	6.69
	β-amyrin 28-oxidase	LOC18783162	1.58	2.32

**Table 2:** Principal pathogenicity-associated genes significantly induced during *M. fructicola* infection of peach fruits. Log_2_FC values at 12 and 24 hpi are reported.

Functional Category	Gene/Protein	Gene ID	log_2_FC (12 hpi)	log_2_FC (24 hpi)
Necrosis induction	Hypersensitive response-inducing protein	EYC84_010656	1.77	3.30
	Necrosis- and ethylene-inducing protein 2 (NEP2)	EYC84_009186	2.07	5.89
	Necrosis-inducing protein (NPP1)	EYC84_006718	1.17	0.58
	Cerato-platanin	EYC84_002901	4.37	8.36
Plant cell wall degradation (CAZymes)	Pectin acetylesterase (CE12)	EYC84_003936	5.49	11.59
	Pectin methylesterase (CE8)	EYC84_010609	2.43	5.50
	Polygalacturonase (GH28)	EYC84_010629	6.21	9.21
	Endo-β-1,4-xylanase (GH11)	EYC84_002893	3.88	4.47
	Acetylxylan esterase/cutinase (CE5)	EYC84_011366	4.80	3.83
	Pectate lyase (PL1)	EYC84_001557	5.28	3.56
	Glycoside oxidoreductase (AA3)	EYC84_005830	5.32	4.59
	Polygalacturonase (GH28)	EYC84_008853	5.21	5.76
	Glycosyltransferase (GT2)	EYC84_004469	3.90	3.25
	Glycoside oxidase (AA7)	EYC84_009707	4.27	3.13
	Lytic polysaccharide monooxygenase (AA9)	EYC84_003941	5.21	4.85
Protein degradation	Aspartic protease	EYC84_011533	6.36	9.12
	Carboxypeptidase cpdS	EYC84_002425	1.99	3.63
	Tripeptidyl peptidase A	EYC84_002157	1.69	4.67
Other candidate effectors/ virulence factors	Egh16-like virulence factor	EYC84_002723	5.84	2.67
	Egh16-like virulence factor	EYC84_007944	5.09	2.21
	Pectin lyase fold protein/virulence factor	EYC84_007698	5.97	2.63
	Chloroperoxidase	EYC84_007503	5.21	6.48
	CVNH (CyanoVirin-N homology)	EYC84_002620	2.59	3.43
	FAS1-domain protein	EYC84_004276	2.98	3.52
	Phosphatidylethanolamine-binding protein	EYC84_003543	2.69	3.29
	RlpA-like double-psi β-barrel	EYC84_011954	2.78	2.99
	Trm112-like protein	EYC84_004931	1.21	2.26
	Long chronological lifespan protein 2	EYC84_005157	1.41	1.28
	Domain involved in innate immunity and lipid metabolism	EYC84_011319	1.87	0.82
	Peptidase S8/Proteinase inhibitor I9	EYC84_005184	1.05	0.19
	Isochorismatase	EYC84_002145	1.73	2.67
	FKBP-type peptidyl-prolyl isomerase	EYC84_006085	0.56	1.06
Stress adaptation	DAP1—damage response protein 1	EYC84_000827	1.26	2.69
	MS95 DDR protein	EYC84_012001	3.64	7.09
	Stress response protein Ish1	EYC84_010183	2.32	4.17
Other pathogenicity-related genes	Cell surface protein	EYC84_001129	2.57	5.76
	Fungal-specific transcription factor	EYC84_002658	1.76	4.12

## Data Availability

The transcriptomic data are publicly available under NCBI BioProject accession number PRJNA1519183. All processed data supporting the conclusions are included in the article and its Supplementary Materials.

## Supplementary Materials

The following supporting information can be downloaded at: https://www.mdpi.com/article/10.3390/jof12100738/s1, Table S1: Lesion diameter during disease progression following *Monilinia fructicola* inoculation of peach fruits; Table S2: RNA-Seq data from peach fruits inoculated or uninoculated with *M. fructicola*; Table S3: Expression levels and differential expression analysis of total genes in *Prunus persica* and *Monilinia fructicola* during infection; Figure S1: *Monilinia fructicola* strain Mfrc123 cultivated on PDA. Figure S2: MapMan overview of transcriptomic changes in *Prunus persica* fruits following *Monilinia fructicola* infection; Table S4: Predicted biosynthetic gene clusters (BGCs) identified in the *Prunus persica* genome using plantiSMASH; Table S5: Predicted biosynthetic gene clusters (BGCs) identified in the *Monilinia fructicola* genome using antiSMASH; Table S6: CAZymes identified by dbCAN analysis in the *M. fructicola* Mfrc123 genome; Table S7: Functionally characterized candidate effector genes of *Monilinia fructicola* significantly upregulated during infection.
