# Supplementary material for: Gene Expression Dynamics in Monilinia fructicola and Prunus persica During the Battle for Infection Revealed by Dual RNA-Seq Analysis
Source: J Fungi (Basel). 2026 Oct 1;12(10):738. doi: 10.3390/jof12100738 (PMC13705664; doi:10.3390/jof12100738)
Supplement: Supplementary file 1 [file jof-12-00738-s001.zip › Dual RNA-Seq supplementary material.pdf]

SUPPLEMENTARY MATERIAL

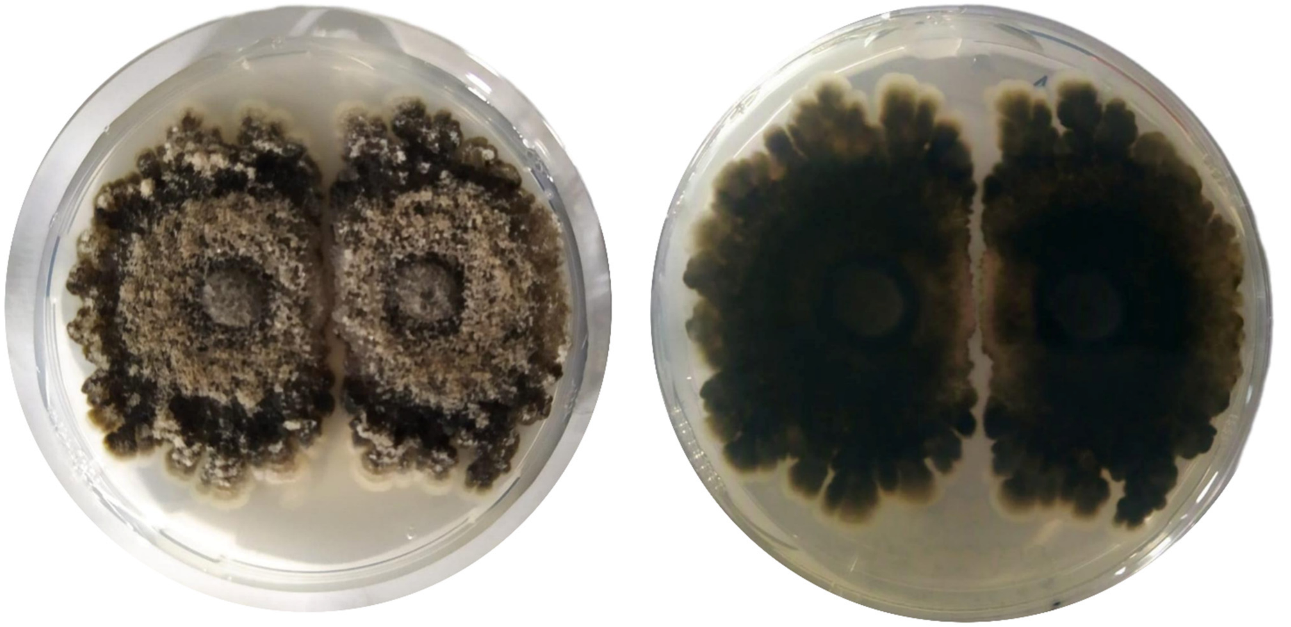

Figure S1. *Monilinia fructicola* strain Mfrc123 cultivated on PDA for 7 days at 25°C in dark conditions.

Table S1. Lesion diameters during disease progression following *M. fructicola* inoculation of peach fruits.

Sampling time	Biological replicate	Lesion diameter (cm)[a]	Average (cm)
0 hpi[b]	1	0.00±0.00	0.00±0.00
	2	0.00±0.00	
	3	0.00±0.00	
12 hpi	1	0.00±0.00	0.00±0.00
	2	0.00±0.00	
	3	0.00±0.00	
24 hpi	1	1.07±0.04	1.03±0.05
	2	0.93±0.02	
	3	1.09±0.05	

[a] Values are the mean of six technical replicates per fruit. Three independent fruits served as biological replicates for each sampling time (n = 3); [b] hpi: hours post-inoculation.

Table S2. RNA-Seq data from peach fruits inoculated or uninoculated with *M. fructicola*.

Experimental condition	Sampling time (hpi[a])	Biological replicate	Total No. reads[b]	Reads mapped (%) on the genomes of		Unmapped reads (%)
				Prunus persica[c]	*Monilinia fructicola*[d]	
Uninoculated fruits	12	1	50,881,012	43,699,600 (85.9)	1,001,742 (2.0)	6,179,670 (12.1)
		2	63,025,916	55,482,380 (88.0)	1,280,430 (2.0)	6,263,106 (10.0)
		3	66,136,708	60,082,080 (90.9)	1,927,680 (2.9)	4,126,948 (6.2)
	24	1	59,261,316	51,840,452 (87.5)	738,592 (1.2)	6,682,272 (11.3)
		2	51,008,918	43,778,274 (85.8)	642,510 (1.3)	6,588,134 (12.9)

		3	59,285,262	50,660,890 (85.5)	1,917,956 (3.2)	6,706,416 (11.3)
Inoculated fruits	0	1	95,377,350	76,283,814 (80.0)	3,353,790 (3.5)	15,739,746 (16.5)
		2	86,829,226	74,815,858 (86.2)	2,892,224 (3.3)	9,121,144 (10.5)
		3	100,483,870	88,483,126 (88.1)	7,130,596 (7.1)	4,870,148 (4.8)
	12	1	65,428,726	57,404,160 (87.7)	2,476,342 (3.8)	5,548,224 (8.5)
		2	66,254,180	58,477,140 (88.3)	2,098,814 (3.2)	5,678,226 (8.5)
		3	60,719,952	54,824,816 (90.3)	2,125,068 (3.5)	3,770,068 (6.2)
	24	1	63,781,460	52,456,098 (82.2)	5,768,228 (9.0)	5,557,134 (8.8)
		2	53,269,360	39,943,528 (75.0)	6,461,670 (12.1)	6,864,162 (12.9)
		3	61,908,712	50,979,102 (82.3)	2,832,324 (4.6)	8,097,286 (13.1)

[a]hpi = hours after inoculation; [b]Total number of 100-bp reads that passed the quality check (QS≥30); [c]*Prunus persica* cv. Lovell, Annotation Release 100, PLov2-2N; GenBank assembly accession GCA_000346465.2; https://www.ncbi.nlm.nih.gov/genome/annotation_euk/Prunus_persica/100); *Monilinia fructicola* strain Mfrc123; GenBank assembly accession GCA_008692225.1; https://www.ncbi.nlm.nih.gov/data-hub/assembly/GCA_008692225.1).

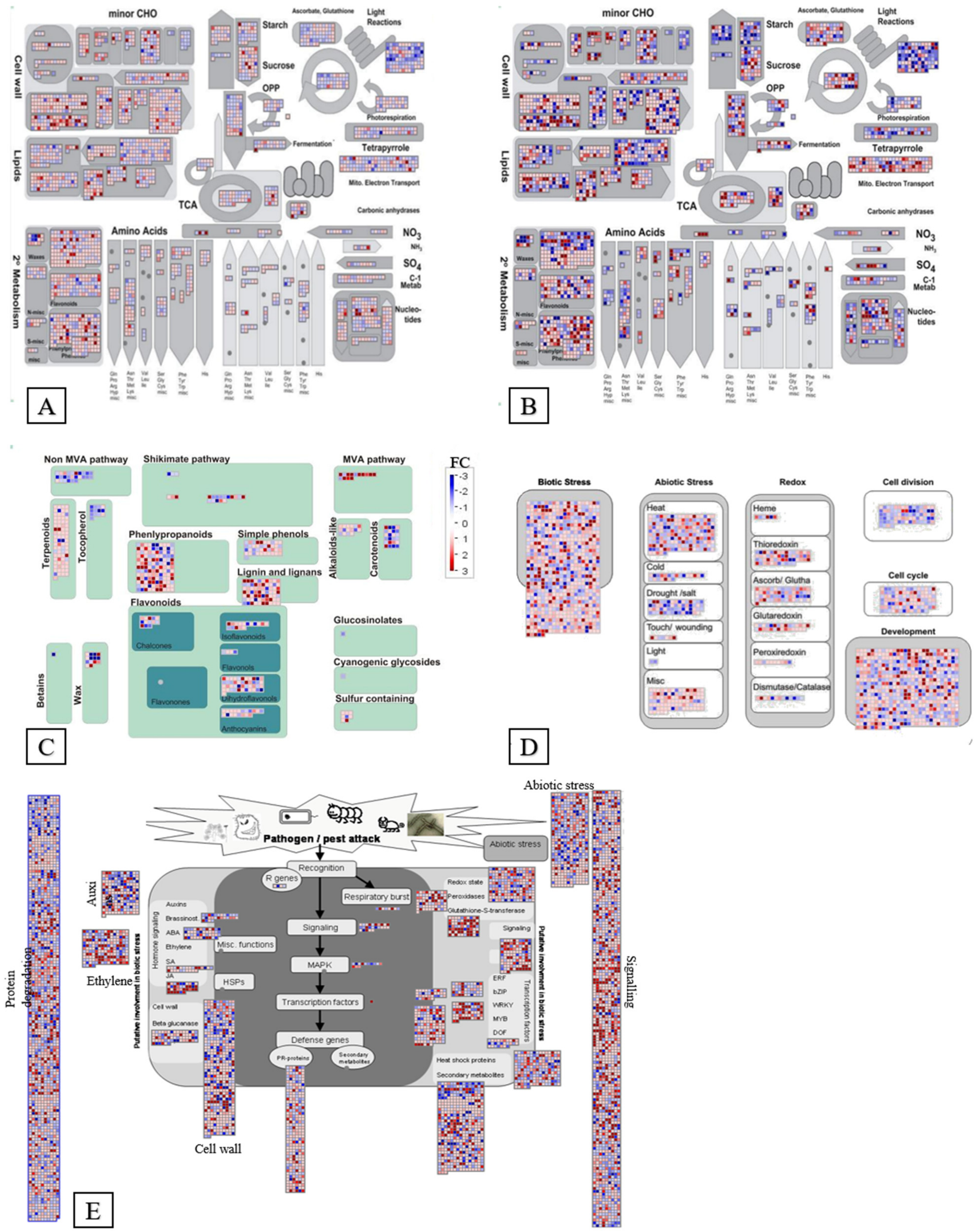

Figure S2. MapMan overview of transcriptomic changes in *Prunus persica* fruits following *Monilinia fructicola* infection. (**A**) Global metabolism overview at 12 hpi; (**B**) global metabolism overview at 24 hpi; (**C**) secondary metabolism at 24 hpi; (**D**) cellular responses at 24 hpi; (**E**) stress-related metabolic pathways at 24 hpi. Gene expression is represented as fold change (FC) between inoculated and uninoculated fruits. The logarithmic colour scale indicates upregulated genes in red and downregulated genes in blue.

Table S4. Predicted biosynthetic gene clusters (BGCs) identified in the *Prunus persica* genome using plantiSMASH.

Cluster	Type	Chromosome	From	To	Size (kb)
Cluster 1	Polyketide - Saccharide	NC_034009.1	248,688.00	378,397.00	129,710
Cluster 2	Putative	NC_034009.1	2,437,013.00	2,588,545.00	151,533
Cluster 3	Cyclopeptide	NC_034009.1	26,334,432.00	26,592,950.00	258,519
Cluster 4	Saccharide	NC_034009.1	36,201,383.00	36,274,825.00	73,443
Cluster 5	Cyclopeptide	NC_034009.1	37,941,087.00	38,324,462.00	383,376
Cluster 6	Saccharide	NC_034009.1	38,347,502.00	38,409,136.00	61,635
Cluster 7	Cyclopeptide	NC_034009.1	38,662,082.00	39,032,025.00	369,944
Cluster 8	Cyclopeptide	NC_034009.1	39,131,270.00	40,050,926.00	919,657
Cluster 9	Polyketide	NC_034009.1	39,203,009.00	39,278,688.00	75,680
Cluster 10	Cyclopeptide	NC_034009.1	39,494,523.00	40,396,804.00	902,282
Cluster 11	Saccharide	NC_034010.1	484,304.00	658,308.00	174,005
Cluster 12	Saccharide	NC_034010.1	21,178,819.00	21,314,420.00	135,602
Cluster 13	Saccharide	NC_034010.1	23,432,590.00	23,737,602.00	305,013
Cluster 14	Polyketide - Lignan	NC_034010.1	25,248,170.00	25,306,630.00	58,461
Cluster 15	Fatty_acid	NC_034010.1	26,550,185.00	26,605,923.00	55,739
Cluster 16	Saccharide	NC_034011.1	928,498.00	1,026,223.00	97,726
Cluster 17	Saccharide	NC_034011.1	19,902,580.00	19,988,821.00	86,242
Cluster 18	Saccharide	NC_034011.1	20,109,574.00	20,358,460.00	248,887
Cluster 19	Terpene	NC_034012.1	1,378,905.00	1,426,776.00	47,872
Cluster 20	Putative	NC_034012.1	7,867,283.00	8,165,677.00	298,395
Cluster 21	Cyclopeptide	NC_034012.1	9,578,787.00	10,185,060.00	606,274
Cluster 22	Saccharide	NC_034012.1	10,239,523.00	10,283,944.00	44,422
Cluster 23	Alkaloid	NC_034012.1	11,209,889.00	11,231,995.00	22,107
Cluster 24	Terpene - Saccharide	NC_034012.1	12,055,517.00	12,494,317.00	438,801
Cluster 25	Saccharide	NC_034012.1	15,156,997.00	15,873,759.00	716,763
Cluster 26	Polyketide	NC_034012.1	16,870,432.00	17,261,414.00	390,983
Cluster 27	Cyclopeptide	NC_034012.1	17,903,870.00	18,627,711.00	723,842
Cluster 28	Saccharide	NC_034013.1	1,744,164.00	1,810,582.00	66,419
Cluster 29	Alkaloid	NC_034013.1	9,096,150.00	9,335,158.00	239,009
Cluster 30	Lignan	NC_034013.1	11,525,557.00	11,570,016.00	44,460
Cluster 31	Cyclopeptide	NC_034013.1	14,773,290.00	15,009,136.00	235,847
Cluster 32	Lignan - Saccharide	NC_034014.1	563,541.00	690,469.00	126,929
Cluster 33	Fatty_acid - Saccharide	NC_034014.1	1,056,146.00	1,141,116.00	84,971
Cluster 34	Saccharide	NC_034014.1	1,316,071.00	1,624,283.00	308,213
Cluster 35	Fatty_acid	NC_034014.1	4,699,067.00	4,812,285.00	113,219
Cluster 36	Saccharide	NC_034015.1	14,273,646.00	14,336,209.00	62,564
Cluster 37	Fatty_acid	NC_034015.1	14,904,691.00	15,025,519.00	120,829
Cluster 38	Putative	NC_034016.1	888,177.00	960,307.00	72,131
Cluster 39	Fatty_acid	NC_034016.1	1,663,535.00	1,787,339.00	123,805
Cluster 40	Polyketide	NC_034016.1	4,185,255.00	4,414,398.00	229,144
Cluster 41	Cyclopeptide	NC_034016.1	12,848,045.00	13,219,811.00	371,767
Cluster 42	Cyclopeptide	NC_034016.1	12,927,603.00	13,324,130.00	396,528
Cluster 43	Saccharide	NC_034016.1	15,283,061.00	15,367,472.00	84,412
Cluster 44	Alkaloid	NC_034016.1	19,667,786.00	19,781,012.00	113,227
Cluster 45	Terpene	NC_034016.1	21,049,780.00	21,078,833.00	29,054

Table S5. Predicted biosynthetic gene clusters (BGCs) identified in the *Monilinia fructicola* genome using antiSMASH.

Cluster	Type	Scaffold	From	To	Size (kb)
Cluster 1.1	NRPS	VICG01000001.1	253,579	316,890	63,312
Cluster 1.2	terpene	VICG01000001.1	980,347	1,012,284	31,938
Cluster 1.3	NRPS	VICG01000001.1	1,535,145	1,597,910	62,766
Cluster 1.4	NRPS	VICG01000001.1	3,407,355	3,478,607	71,253
Cluster 2.1	T1PKS	VICG01000002.1	2,004,780	2,070,768	65,989
Cluster 3.1	terpene	VICG01000003.1	279,061	311,420	32,360
Cluster 3.2	NRPS	VICG01000003.1	779,936	848,921	68,986
Cluster 3.3	NRPS-like, T1PKS	VICG01000003.1	1,734,135	1,860,595	126,461
Cluster 3.4	terpene	VICG01000003.1	2,007,845	2,040,612	32,768
Cluster 3.5	terpene	VICG01000003.1	2,870,023	2,903,643	33,621
Cluster 4.1	NRPS-like	VICG01000004.1	30,017	94,282	64,266
Cluster 4.2	NRPS-like	VICG01000004.1	517,385	588,877	71,493
Cluster 5.1	NRPS-like	VICG01000005.1	1,078,167	1,142,221	64,055
Cluster 6.1	NRP-metallophore, NRPS	VICG01000006.1	190,716	269,337	78,622
Cluster 6.2	NRPS-like, NRPS	VICG01000006.1	1,512,088	1,606,637	94,550
Cluster 6.3	T1PKS	VICG01000006.1	2,183,515	2,251,983	68,469
Cluster 7.1	T1PKS	VICG01000007.1	88,576	170,174	81,599
Cluster 7.2	other	VICG01000007.1	454,817	515,755	60,939
Cluster 7.3	terpene	VICG01000007.1	1,066,650	1,098,266	31,617
Cluster 8.1	terpene	VICG01000008.1	626,991	658,575	31,585
Cluster 8.2	T1PKS	VICG01000008.1	1,832,822	1,899,566	66,745
Cluster 9.1	NRPS	VICG01000009.1	2,066,019	2,135,938	69,920
Cluster 11.1	NRPS-like	VICG01000011.1	1,040,754	1,104,089	63,336
Cluster 11.2	T1PKS	VICG01000011.1	1,546,623	1,614,104	67,482
Cluster 11.3	T1PKS	VICG01000011.1	2,057,176	2,124,755	67,580
Cluster 11.4	terpene	VICG01000011.1	2,311,241	2,335,009	23,769
Cluster 12.1	T1PKS	VICG01000012.1	176,733	244,723	67,991
Cluster 12.2	T1PKS	VICG01000012.1	1,581,361	1,648,261	66,901
Cluster 13.1	indole	VICG01000013.1	30,030	61,421	31,392
Cluster 13.2	T1PKS	VICG01000013.1	91,488	159,654	68,167
Cluster 13.3	T1PKS	VICG01000013.1	371,910	439,480	67,571
Cluster 13.4	T3PKS	VICG01000013.1	700,858	762,524	61,667
Cluster 14.1	terpene-precursor	VICG01000014.1	147,636	178,877	31,242
Cluster 15.1	T1PKS	VICG01000015.1	1	44,817	44,817
Cluster 15.2	terpene-precursor	VICG01000015.1	1,107,664	1,138,936	31,273
Cluster 16.1	T1PKS	VICG01000016.1	80,684	151,310	70,627

Table S7. Functionally characterized candidate effector genes of *Monilinia fructicola* significantly upregulated during infection (FC ≥ 2 and FDR ≤ 0.05). Fold change values at 12 and 24 hpi, and EffectorP predictions are reported.

Gene ID	Functional domains	EP prediction	Prediction EffectorP 3.0	Accuracy (%)	FC (12hpi)	FC (24hpi)
EYC84_007698	Pectin lyase fold/virulence factor	Ep3	Apoplastic effector	60.7	62.60	6.20
EYC84_002723	Egh16-like virulence factor	Ep3	Apoplastic effector	57.3	57.29	6.38
EYC84_007503	Heme haloperoxidase domain-containing protein; cloroperoxidase	Ep3	Non-effector	67.3	37.13	89.49
EYC84_008853	Glycoside hydrolase, family; pectin lyase fold/virulence factor	Ep2	Apoplastic effector	60.6	37.09	54.01
EYC84_007944	Egh16-like virulence factor	Ep3	Cytoplasmic effector	50.8	34.04	4.62
EYC84_007617	Tuberculosis necrotizing toxin	Ep3	Apoplastic effector	63.9	30.51	7.21
EYC84_002901	Cerato-platanin	Ep3	Cytoplasmic effector	68.2	20.62	328.54
EYC84_004276	FAS1 domain superfamily	Ep3	Cytoplasmic effector	73.1	7.87	11.45
EYC84_011954	RlpA like double-psi beta-barrel	Ep1, Ep2, Ep3	Non-effector	54.2	6.85	7.92
EYC84_003543	Phosphatidylethanolamine-binding protein	Ep1, Ep3	Cytoplasmic effector	77.5	6.45	9.80
EYC84_002620	CVNH - CyanoVirin-N homology	Ep3	Apoplastic effector	85.9	6.04	10.76
EYC84_009186	Necrosis inducing protein	Ep2, Ep3	Apoplastic effector	79.8	4.20	59.45
EYC84_011319	Domain involved in innate immunity and lipid metabolism	Ep3	Cytoplasmic/apoplastic effector	61.5	3.65	1.76
EYC84_002145	Isochorismatase	Ep3	Non-effector	76.6	3.31	6.35
EYC84_005157	Long chronological lifespan protein 2	Ep1, Ep2, Ep3	Cytoplasmic effector	86	2.65	2.44
EYC84_004931	Trm112p-like protein	Ep3	Cytoplasmic effector	75.1	2.31	4.77
EYC84_006718	Necrosis inducing protein, NPP1	Ep3	Apoplastic effector	72.4	2.24	1.49
EYC84_005184	Peptidase S8 propeptide/proteinase inhibitor I9 superfamily	Ep1, Ep2	Cytoplasmic effector	64.7	2.07	1.14
EYC84_006085	FKBP-type peptidyl-prolyl cis-trans isomerase domain	Ep3	Cytoplasmic effector	89.2	1.48	2.08
